# Computational method to analyze linear developmental gradients reveals specific metabolite enrichment patterns in stress-tolerant maize

**DOI:** 10.1242/dev.205350

**Published:** 2026-05-07

**Authors:** Andrea M. Sama, Sinead B. Cahill, Shihong Luo, Abigail L. Tripka, Yifan Meng, Sarah E. Noll, Richard N. Zare, Pavak Shah, Alexandra Jazz Dickinson

**Affiliations:** ^1^ Department of Cell and Developmental Biology, UC San Diego, La Jolla, CA 92102, USA; ^2^ Department of Chemistry, UC San Diego, La Jolla, CA 92102, USA; ^3^ Department of Chemistry, Stanford, Stanford, CA 94305, USA; ^4^ Department of Chemistry at Pomona College, Claremont, CA 91711, USA; ^5^ Molecular, Cell, and Developmental Biology Department, UC Los Angeles, Los Angeles, CA 90095, USA; ^6^ Institute for Quantitative and Computational Biosciences, UC Los Angeles, Los Angeles, CA 90095, USA; ^7^ Soil Health Center, Scripps Institution of Oceanography, La Jolla, CA 92093, USA

**Keywords:** Root development, Cell differentiation, Stress resilience, Spatial metabolomics, Bioinformatics, Erythrose

## Abstract

Metabolic processes are essential for regulating and maintaining developmental transitions. However, the distinct metabolite-driven mechanisms that are crucial for development remain poorly characterized due to inherent challenges in measuring their localization and function *in situ*. We applied desorption electrospray ionization mass spectrometry imaging (DESI-MSI) to generate near single-cell resolution (50-80 µm) images of metabolites in the maize root tip, which has a well-characterized longitudinal developmental gradient. We developed a new computational tool, called Developmental Imaging Mass Spectrometry Pipeline for Linear Evaluation (DIMPLE), which processes mass signatures along linear gradients and clusters metabolites based on their developmental enrichment patterns. We employed this method to compare developmental enrichment of metabolites in Oaxacan Green, a salt-resilient maize variety, to B73, which is salt sensitive. DIMPLE uncovers specific differences in individual mass signatures and overall enrichment patterns between these varieties. Further characterization of these differences revealed meristem enrichment of D-erythrose, a metabolite that can improve stress tolerance in maize. Overall, DIMPLE enables comprehensive and rapid analysis of metabolite patterns along a linear gradient, informing biological hypotheses related to plant growth and stress response.

## Teaser

**Summary:** DIMPLE, a hypothesis-driven approach to biostatistical analysis of spatial ‘omics data, is used to characterize the chemical biology of development and stress tolerance in maize roots.

## INTRODUCTION

Distinct metabolic processes are essential for developmental transitions between cell stages such as stem cell quiescence, proliferation and differentiation. Each developmental transition requires specific cellular energetic states as well as metabolite-driven signaling and protein interactions. In plants, metabolites play important roles as regulators that bridge development and environmental responses (Erb and Kliebenstein, 2020). For example, a classic control point regulating both development and stress responses in plants is the phytohormone abscisic acid, which is activated by abiotic stress and regulates seed germination and root growth (Hauser et al., 2017; Nakashima and Yamaguchi-Shinozaki, 2013; Tuteja, 2007). However, despite the importance of metabolites in development and stress responses, metabolite-based mechanisms remain poorly characterized due to inherent challenges in measuring their production, localization and function *in situ*. These problems are amplified when considering metabolites with precise spatial enrichment patterns, such as those that occur during developmental transitions. Metabolomics approaches typically require relatively large amounts of tissue (mg to g scale), masking the metabolic signatures of rare cell types, such as stem cells (Jackson and Finley, 2024). Current approaches to study metabolites in rare cell types often require these cells to be physically isolated from the tissue (Binek et al., 2019; Jackson and Finley, 2024). However, these approaches can influence the cell metabolome (Binek et al., 2019) and isolation of cells can remove microenvironment cues (Jackson and Finley, 2024), which may be crucial, particularly during development. Additionally, bulk tissue approaches do not capture the heterogeneity of developing cells, which may be in a variety of transitional states in terms of differentiation, quiescence and stress response (Miyazawa and Aulehla, 2018).

Mass spectrometry imaging (MSI) offers an alternative method of approaching metabolomic investigations in developing tissue (Gao et al., 2023; Horn et al., 2012; Korte et al., 2015; Kumara et al., 2016). There are many approaches to MSI, such as desorption electrospray ionization (DESI), matrix assisted laser desorption ionization (MALDI) and secondary ion mass spectrometry (SIMS). These different techniques have their own advantages and limitations, and have been thoroughly reviewed (Buchberger et al., 2018; Gao et al., 2023; Prentice and Caprioli, 2016). MSI provides *in situ* analysis of the chemical makeup of tissue sections with high spatial resolution ranging from ∼5 to 100 µm depending on the ionization method. This can enable near single-cell visualization of metabolomic changes throughout tissue, which is advantageous for studying rare cell types (Jun et al., 2010; Lanni et al., 2012; Römpp and Spengler, 2013). Furthermore, imaging cells in their native microenvironment maintains the structure of tissue and the cues that regulate developmental transitions.

To investigate metabolite enrichment patterns throughout development, we utilized DESI-MSI to map the chemistry of the developing maize root (Zhang et al., 2023b). DESI-MSI uses a charged solvent spray to desorb compounds from the surface of the tissue. The desorbed analytes are then ionized in secondary droplets that are detected by the mass spectrometer. Moving the desorption spot along the tissue's surface generates a rasterized image, in which every pixel contains a full mass spectrum. By collecting full mass spectra, the varying intensities of individual molecular mass signatures can be mapped throughout the tissue, providing spatial information for a range of metabolites, lipids and peptides. This information can be used to predict the identity and function of endogenous chemical compounds.

**Fig. 1.**
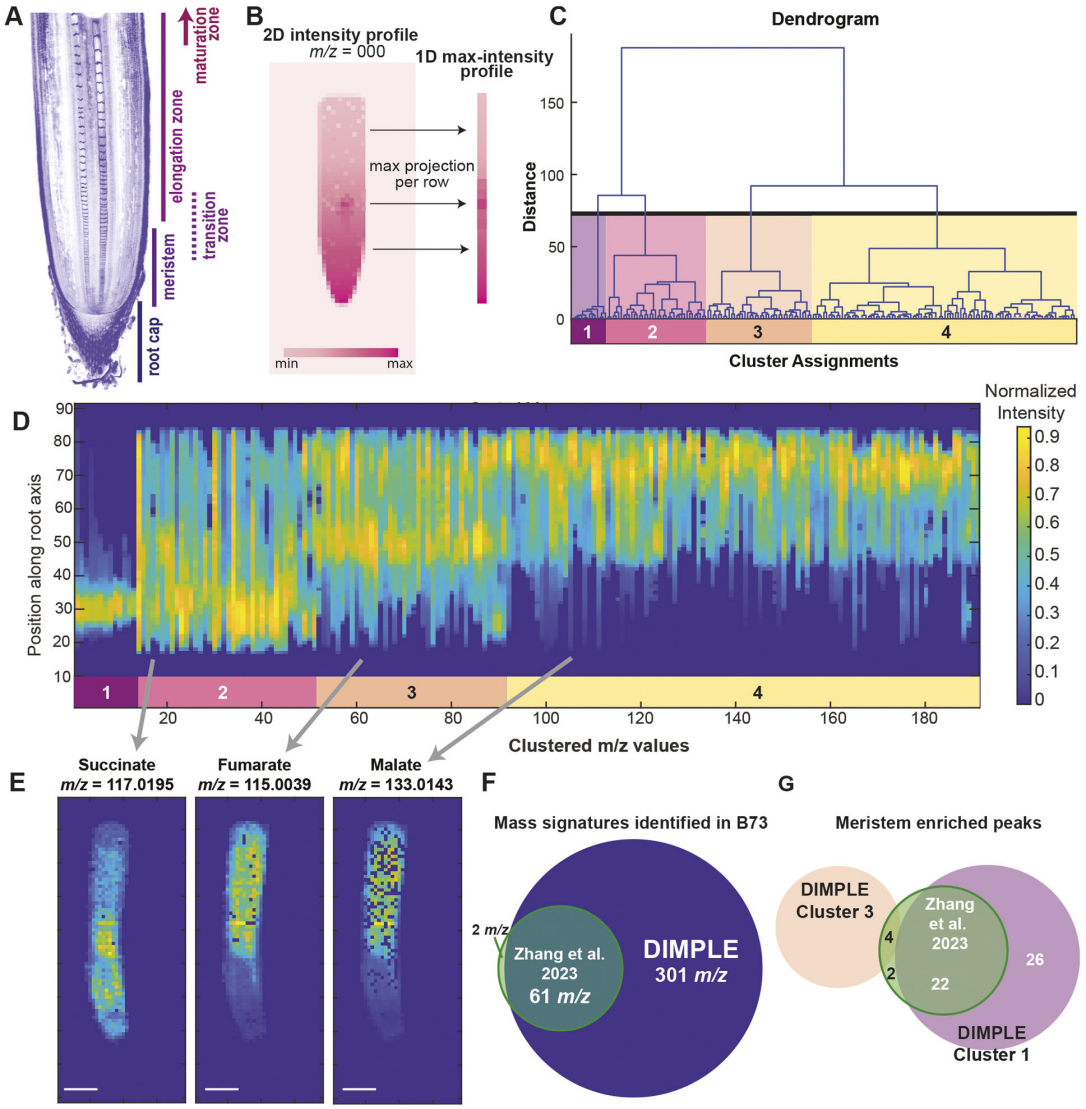
**DIMPLE method increases *m/z* identification.** (A) Confocal image of a maize root section stained with Calcofluor White, visualizing the developmental axis characterized by the root cap, meristem, transition zone, elongation zone and maturation zone. (B) Schematic of the DIMPLE approach, reducing the 2D intensity profile of the MSI data into a 1D linescan profile, based on the maximum projection per row. (C) Ward's hierarchical clustering method results in clusters corresponding to spatial similarity of linescan intensity profiles. User-defined number of clusters can then be visualized as shown in D. (D) Linescan graph showing 1D intensity profiles of the mass signatures in each cluster. Clusters are designated by the colored bars arranged left to right and labeled with corresponding cluster number. The *y*-axis is the position along the root axis (pixels) where 0 is closest to the meristem and 90 is the mature root tissue. The *x*-axis corresponds to the clustered *m/z* linescans arranged smallest to largest within each cluster. The intensity of each linescan is normalized to a maximum value of 1, as represented in the ‘Normalized Intensity’ key. (E) 2D spatial maps of the signal enrichment for succinate (*m/z* 117.0195), fumarate (*m/z* 115.0039) and malate (*m/z* 133.0143) in a B73 maize root. 2D images were generated using the DIMPLE GUI. Scale bars: 10 pixels. (F) The DIMPLE method identified 301 unique *m/z* values compared to the 63 in the Zhang et al. (2023b) paper. For the full list of values, see Tables S1 and S2. (G) In the DIMPLE analysis, 48 mass signatures grouped to Cluster 1 across three B73 replicates, whereas 28 root tip-enriched metabolites were identified by Zhang et al. (2023b). Twenty-two of the previously identified peaks were included in Cluster 1, four were grouped to Cluster 3 (see Fig. S4) and two were not identified by DIMPLE. Images and peak lists can be found in Figs S3 and S4 and Table S3.

In a previous study, we applied this technique to longitudinal cryosections of maize roots at a spatial resolution of approximately 80 µm (Zhang et al., 2023b). Plant roots represent a highly stereotyped developmental gradient (Fig. 1A). At the root tip is the meristem region, which encompasses the stem cell niche and dividing pluripotent cells. Progressing along the root axis, cells lose their ability to divide and begin to lengthen in the elongation zone (EZ). These developmental transitions are encompassed in a ∼5 mm longitudinal section of the maize root tip (Alarcón et al., 2014). Cells finally transition to full maturity in the differentiation zone (Hochholdinger, 2009; Marcon et al., 2015). Our previous work applying DESI-MSI to the maize root tip identified 63 mass signatures with specific localization patterns along this developmental axis (Zhang et al., 2023b). One finding from this work was that tricarboxylic acid (TCA) cycle metabolites have distinct localization patterns across the developmental gradient of the root. For instance, of all the TCA cycle metabolites we measured, succinate was the only one enriched in the meristem region of maize roots. Further study of this specificity revealed that exogenous succinate treatment increased meristem cell divisions in *Arabidopsis* roots, demonstrating that localization patterns can inform predictions of developmentally relevant metabolite functions (Zhang et al., 2023b).

Although MSI has enormous potential as a tool to characterize metabolites in developing tissue, there are challenges to analyzing and interpreting MSI datasets. MSI datasets are large; each pixel contains a full mass spectrum with hundreds to thousands of peaks. This sheer volume of data presents a challenge, especially when conducting an untargeted analysis of chemical distributions throughout tissues. Several commercial and open-source software options have been developed to aid in the processing and analysis of MSI data (Bemis et al., 2015; Bokhart et al., 2018; Hu and Laskin, 2022; Källback et al., 2016; Robichaud et al., 2013). A common software, previously open-source and now commercial, used for MSI data analysis is MSiReader (Bokhart et al., 2018; Robichaud et al., 2013). MSiReader v1.03 allows users to optimize visualization of *m/z* features by adjusting parameters such as normalization method, image overlay, and colocalization of metabolite signals. MSiReader v1.03 also provides several tools to aid in the analysis of untargeted metabolomics, such as MSi Correlation and MSi Peakfinder strategies. However, these methods rely on user-defined regions of interest or peak selections and the processing times for these analyses can be significant. MSiReader v1.03 includes a plug-in to METASPACE that enables database searching to annotate unknown mass signatures. METASPACE is an open-source cloud platform for spatial data interpretation and metabolite identification (Palmer et al., 2017). It performs automated, false discovery rate-controlled metabolite identification using an array of user-selected databases, creates visualization profiles of specific annotations, and compares localization patterns across tissues. Other mass spectrometry resources, such as Metlin Classic and LOTUS, can also be useful in annotating mass signatures. Metlin Classic is a metabolite search engine that provides hits for *m/z* values based on ionization mode and defined mass error; it also includes fragmentation patterns for additional confirmation (Guijas et al., 2018; Smith et al., 2005). LOTUS is an open-source tool for natural products identification and is particularly helpful for annotating plant metabolites (Rutz et al., 2022). These resources were all valuable in identifying molecular patterns of interest and/or predicting the chemical identities of *m/z* signatures in our datasets.

We applied many of these resources to analyze our maize root DESI-MSI data and found dozens of mass signatures with distinct patterns during development (Zhang et al., 2023b). However, these identifications required a labor-intensive manual interpretation of the raw data and sorting of developmental patterns, in addition to the utilization of open-source software. This work revealed the need for a new computational method to identify mass signatures along the linear root developmental gradient. To address this gap and streamline untargeted analysis, we developed a tool specifically for interpretation of plant root MSI data. MSI analytical tools typically include four main steps: (1) pre-processing, (2) peak picking, (3) de-noising and (4) spatial clustering (Alexandrov et al., 2010, 2013; Alexandrov and Kobarg, 2011; Ovchinnikova et al., 2020). There are several approaches that utilize variations of these steps to identify peaks and cluster them based on measures of 2D spatial similarity (Alexandrov et al., 2010; Zhang et al., 2023c). While unbiased spatial clustering is broadly applicable to many types of tissue, we sought to reveal patterns based on a specific spatial prediction – that the linear enrichment of a molecule along the developmental gradient would reveal useful biological information. Therefore, we created a computational method called Developmental Imaging Mass Spectrometry Pipeline for Linear Evaluation (DIMPLE), which is designed to leverage the linear developmental axis of plant roots to reveal distinct metabolite patterns that correspond to the transitions between quiescence, proliferation, elongation and differentiation. We validated its performance and utilized DIMPLE to compare developmental patterns in a stress-tolerant maize variety compared to a stress-susceptible variety. DIMPLE led to the detection of a meristem- and EZ-localized metabolite, D-erythrose, which, in salt-sensitive maize varieties, improves root growth during stress.

Overall, DIMPLE contributes to the growing number of tools used for MSI analysis. It provides a simple and effective approach to cluster mass signatures along linear developmental gradients, allowing for characterization of root metabolite patterns to inform biological hypotheses regarding developmental significance.

## RESULTS AND DISCUSSION

### Development and implementation of DIMPLE

The DIMPLE workflow consists of two core steps. First, raw DESI-MSI data is pre-processed to identify a set of consensus peaks and their corresponding *m/z* values across all pixels; then, observed peaks across all acquired spectra are assigned to the *m/z* values identified in the consensus set. Second, these harmonized spectra are converted to a hyperspectral image that can be analyzed using conventional image analysis and signal processing approaches to identify linear patterns of enrichment along the primary tissue axis. Pre-processing consists of the following steps: (1) optional hot pixel detection and suppression, (2) manual segmentation of the sample boundary from background, (3) generation of a pseudobulk spectrum by summing all peaks detected in every pixel, (4) identification of consensus peaks in the pseudobulk spectrum by identifying local maxima within a ±2.5 ppm window, (5) suppression of peaks that are not enriched in the sample over background, (6) assignment of every observed sample-enriched peak in each pixel to the peak centers identified in the consensus spectrum, (7) reorganization of the harmonized spectra into a hyperspectral image, and (8) removal of peaks that show sparse localization. The removed peaks are retained separately and can be manually inspected for any potential patterns that were erroneously removed. This workflow is designed to ensure that all observed peaks in the sample are aligned to each other independent of instrument jitter within a reasonable error window.

Following this preprocessing, we devised a simple strategy for the unbiased discovery of spatial motifs in metabolite enrichment in the maize root. We focused specifically on the analysis of patterns along the longitudinal (developmental) axis of the root, as the development and growth of the root along this axis is well characterized (Fig. 1A). Adopting a standardized orientation of the longitudinal axis of the root along the *y*-axis of the image, we computed a maximum projection of the 2D hyperspectral image generated by the preprocessing step along the *x*-axis, reducing each image to a 1D longitudinal linescan (Fig. 1B). Each linescan was normalized such that its maximum value is 1 and smoothed with a 10-pixel wide median filter. We then performed hierarchical clustering of these linescans, each corresponding to an *m/z* value, using the Euclidean norm between linescans as the distance metric (Fig. 1C). A common limitation to hierarchical clustering with MSI data is the amount of computational memory and power required to process the linkages between spectral patterns (Alexandrov and Kobarg, 2011; Deininger et al., 2008). Since our method reduces the spectral patterns to a single pixel-wide (one-dimensional) linescan across a consensus set of high-quality channels, data volume is reduced drastically, resulting in a less-intensive processing for hierarchical clustering. By using Ward's linkage method, each spectral linescan is first considered its own cluster, and are sequentially merged using a bottom-up approach whereby patterns are merged to preference the smallest within cluster variances. For each dataset, we determined an appropriate number of clusters that encompass general developmental patterns by examining the structure of the resulting dendrograms (Fig. 1C, Fig. S1) and by examining the distributions of localization patterns among clusters (Fig. 1D). We implemented a simple GUI in MATLAB that allows the user to browse the clustered linescan data and extract the original 2D image for any corresponding channel for export (Fig. 1E).

To confirm that DIMPLE can recover previously identified peaks from our datasets, we compared the localization patterns of several high enrichment metabolites from the DIMPLE GUI to MSiReader-generated images. Specifically, we imaged malate, succinate and fumarate, which have distinct localization patterns, and are represented in all the samples we have previously analyzed (Fig. 1E) (Zhang et al., 2023b). We found that DIMPLE generated images similar to those obtained with MSiReader (Fig. 1E, Fig. S2). The main differences are that the DIMPLE images are not normalized and tend to have more empty pixels, which are the result of background suppression steps during analysis of the raw spectrum.

The enrichment patterns represented in each cluster generally correspond to known developmental domains along the root axis. The clusters we generated for B73 maize roots encompass four metabolite distribution patterns, which we term Clusters 1-4: Cluster 1, strong meristem enrichment; Cluster 2, distribution throughout the meristem and transition zone (TZ); Cluster 3, peak enrichment in the TZ and EZ; Cluster 4, strong enrichment in non-meristematic, differentiating tissue. Of note is the varied enrichment patterns found in Cluster 2, with approximately 40-78% of the signatures in this cluster showing characteristics of noise upon visual inspection of the 2D images (Fig. 1D, Fig. S1).

Overall, DIMPLE enables the visualization of hundreds of *m/z* signatures throughout the developmental gradient. It additionally provides information on how metabolites are related to one another through hierarchical clustering of the linear patterns of enrichment along the root's developmental axis. This provides a useful approach to identify uncharacterized compounds with specific developmental localization patterns.

### DIMPLE identifies metabolites with distinct localization patterns

To validate DIMPLE's clustering and utility, we applied it to previously published data sets in B73 maize roots. DIMPLE was able to identify an average of 196 *m/z* signatures per root (301 unique *m/z* values were identified across three replicates; Tables S1 and S2). In the original paper, 63 peaks were included, which were manually curated and excluded noise (Fig. 1F, Table S1). DIMPLE identified 97% of the *m/z* signatures published in the Zhang et al. (2023b) paper. The two signatures that were not identified by DIMPLE in the final processed lists were either at very low abundance in the replicates investigated here or were only detected in a small number of pixels and thus removed by the image sparsity filter. We also used DIMPLE to map previously uncharacterized mass signatures localized to specific regions of the root. DIMPLE assigned 48 mass signatures to Cluster 1 across three B73 replicates, excluding peaks that were likely noise upon 2D visualization. By contrast, 28 meristem- and root tip-enriched mass signatures were identified by Zhang et al. (2023b). Of the 28 mass signatures previously identified, 22 were included in Cluster 1 peaks, four were grouped to Cluster 3 peaks, and two were not pulled out by DIMPLE (Fig. 1G, Table S3). DIMPLE provided 26 new root tip mass signatures to characterize (Table S3, Fig. S3).

Notably, more signatures can be identified by relaxing parameters such as the ppm tolerance (increasing the ppm threshold from 2.5 to 5 ppm increases the average by 14%, to 223 *m/z* peaks per B73 root) or the minimum threshold enrichment compared to background (decreasing this to 1.1 from 1.5 slightly increases the average by 3%, to 199 *m/z* peaks per B73 root). Relaxing these parameters can provide more targets but careful analysis is required to identify any false signal or noise selected by running a less-stringent analysis. Additionally, including the sparse signal removed peaks can result in a >10-fold increase in the number of peaks identified. However, unless the goal of analysis is to generate a completely comprehensive list of signals from the MSI run, it is not recommended that the sparse signal removal step be skipped, as many of these peaks may be noise or overlapping signals for the same mass signature.

**Fig. 2.**
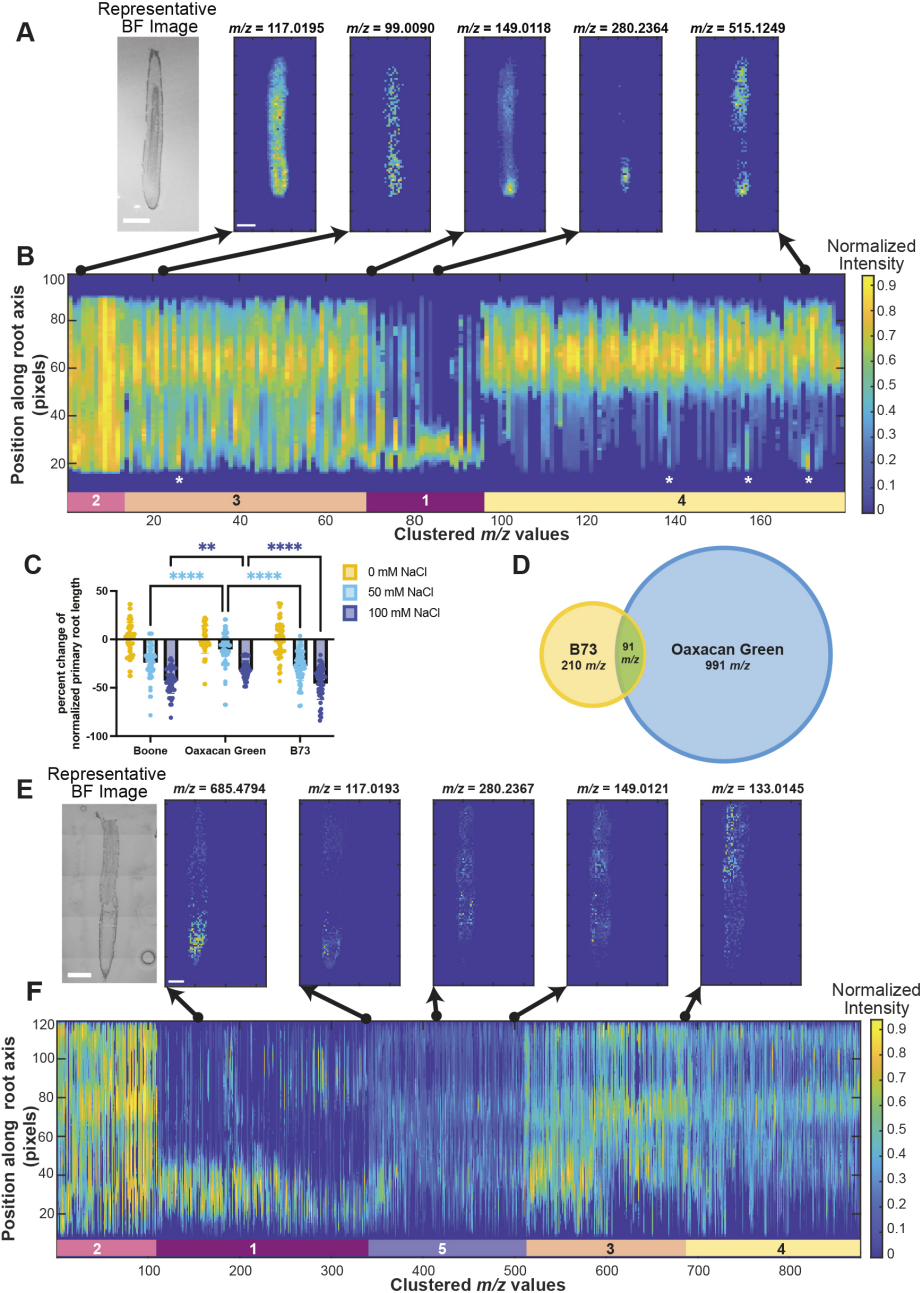
**DIMPLE identifies key developmental differences within and between maize varieties.** (A) From left to right: Representative brightfield (BF) image of B73 maize cryosection (root 1) used for DESI-MSI, and the DIMPLE-generated images for *m/z* 117.0195 (succinate), *m/z* 99.0090, *m/z* 149.0118, *m/z* 280.2364 and *m/z* 515.1249. Images were taken at approximately 80 µm resolution. Scale bars: 1 mm (BF image); 10 pixels (DIMPLE images). (B) Linescan graph for the 1D intensity profiles of mass signatures identified in a B73 maize root. Clusters are designated by the colored bars and labeled with the corresponding cluster number. The *y*-axis corresponds to position along the root axis (pixels) where signal closest to 0 is the root tip and signal closest to 100 corresponds to the mature root tissue. The *x*-axis represents the clustered *m/z* linescans arranged smallest to largest *m/z* value within each cluster. The arrows point to the 2D image in A that corresponds to that position in the linescan graph. Asterisks denote observed areas with bimodal patterns in the linescan. The intensity is normalized to 1. Intensity value is represented according to the colored ‘Normalized Intensity’ key. (C) Percentage change of normalized primary root length for Oaxacan Green, Boone and B73 maize under control, 50 mM and 100 mM NaCl conditions. ****P<0.0001, **P<0.005 (ordinary two-way ANOVA, Tukey's multiple comparisons test, with a single pooled variance). Data reported as mean±s.d. Sample size for all varieties was *n*=60 for 0 mM and 50 mM NaCl conditions, and *n*=48 for 100 mM NaCl conditions. (D) DIMPLE identified 301 unique mass signatures in three B73 root replicates, and 1082 unique mass signatures in three Oaxacan Green replicates. Ninety-one of these mass signatures were conserved between the two varieties. For full peak lists, see Tables S2, S8 and S9. (E) From left to right: Representative BF image of an Oaxacan Green root cryosection (root 1) used for DESI-MSI, and DIMPLE-generated images for *m/z* 685.4794, *m/z* 117.0193 (succinate), *m/z* 280.2367, *m/z* 149.0121 and *m/z* 133.0145 (malate). Images were taken at approximately 50 µm resolution. Scale bars: 1 mm (BF image); 10 pixels (DIMPLE images). (F) Linescan graph of the 1D intensity profiles of mass signatures identified in an Oaxacan Green root. Clusters are designated by the colored bars and labeled with the corresponding cluster number. The *y*-axis corresponds to position along the root axis (pixels) with 0 closest to the meristem and 120 closest to the mature root tissue. The *x*-axis corresponds to clustered *m/z* linescans arranged largest to smallest *m/z* value within each cluster. The intensity is normalized to 1. Intensity value is represented according to the colored ‘Normalized Intensity’ key.

Our analysis with DIMPLE resulted in the discovery of new enrichment patterns along the developmental axis. For example, the peaks in Cluster 1 of B73 roots (Fig. 2A,B) have high enrichment in the root tip and the meristem. Yet, as evidenced by the four root tip-localized peaks identified by Zhang et al. that were associated with a different cluster, the DIMPLE clustering method can provide a more comprehensive analysis of localization patterns. For example, both *m/z* 149.0118 and succinate (*m/z* 117.0195) were manually assigned as root tip- and/or meristem-enriched compounds (Zhang et al., 2023b). However, while DIMPLE consistently clustered *m/z* 149.0118 with other metabolites that are strongly enriched in the root tip (Cluster 1) (Fig. 2A,B, Tables S4-S6), succinate was assigned to Cluster 2 in two of our replicates, and Cluster 1 in one replicate (Figs 2A,B and 1D,E, Figs S1 and S2, Tables S4-S6). When comparing the metabolite localization of succinate and *m/z* 149.0118, succinate was more enriched throughout the developmental axis, while *m/z* 149.0118 was highly specific to the root tip region (Fig. 2A). DIMPLE can parse these differences and this specificity in localization characterization may help to better identify compounds that are linked in developmental function through shared enrichment. In addition to succinate, several other metabolites previously characterized as root tip and/or meristem enriched, such as a molecule predicted to be succinic anhydride (*m/z* 99.0090), showed enrichment along the transition from the root tip to the meristem and EZ (Fig. S4), and were clustered correspondingly to Cluster 3 instead of Cluster 1. This indicates that, like the succinate pattern, while these mass signatures have enrichment in the meristem, their distribution patterns are more closely related with peaks concentrated in the region of transition between the meristem and EZ, based on the hierarchical clustering method (Fig. 1C, Figs S1 and S4). This highlights the more nuanced and holistic approach that DIMPLE provides for mapping the chemistry of developmental transitions, as well as the importance of investigating enrichment patterns in all clusters to identify compounds of interest.

Building off the observation that meristem-enriched metabolites could be grouped outside of the expected meristem-enriched cluster (Cluster 1), we identified several bimodally distributed metabolite patterns. Clusters 3 and 4 had several *m/z* signatures with enrichment in the meristem region, decreased signal in the TZ, and then strong signal again in the maturing tissue (Fig. 2A,B, asterisks, Fig. S5, Table S7). Scanning through these clusters with the GUI identified at least 15 signatures with distinct bimodal distributions, defined by enrichment in the meristem and then again in the mature elongation region (Fig. 2A, Fig. S5, Table S7). Of note, not all *m/z* features showed a bimodal pattern across all three replicates (Table S7), which could be due to differences in sectioning, imaging artifacts or biological variation. However, this is an interesting developmental pattern as it might be expected that the metabolic demands of stem cells and differentiating tissue would be distinct. Further investigation into the identity of these bimodal metabolites, especially those conserved across multiple replicates, could reveal new understanding of metabolite-driven regulation of distinct developmental stages.

In addition to revealing more nuanced developmental patterns in the meristem region, DIMPLE also facilitates analyses of distinct patterns during differentiation. An observation in the supplement of the Zhang et al. (2023b) publication was that several mass signatures seemed to be localized to specific cell layers of the root axis, such as the cortex or vasculature. While DIMPLE condenses 2D information into a 1D linescan to perform the clustering, the DIMPLE GUI recovers the 2D information by visualizing the chemical map corresponding to a selected feature in the linescan. Using the DIMPLE GUI to assess the localization patterns within clusters, we identified more than 15 mass signatures in Cluster 4 of B73 roots with specific enrichment in the cortex (Fig. S6, Table S7), and more than ten mass signatures with enrichment in the vasculature (Fig. S7, Table S7). This demonstrates that DIMPLE can be used to investigate variations within developmental localization clusters and further provide uncharacterized patterns for future investigation.

DIMPLE allows for a more comprehensive analysis of untargeted MSI data. The GUI function can scan through multiple biological replicates, enabling the identification of instrumentation or biological variation between samples that can inform hypotheses on conserved developmental localization. We have successfully used this method to identify dozens of new metabolite candidates with developmentally specific patterns that have exciting potential for uncovering novel biological functions. Overall, this demonstrates that DIMPLE is a useful tool for approaching the untargeted analysis of MSI data and can successfully cluster mass signatures along the developmental regions of the root axis.

### Comparing metabolite localization and distribution across maize varieties with different stress tolerances

The ability to cluster MSI data based on developmentally relevant patterns increases the speed and depth of our analysis pipeline. To utilize these advantages to uncover new biology, we sought to compare developmental chemistry across different maize genotypes. Specifically, we wanted to understand how the chemistry along the root developmental gradient differs between stress-resistant and stress-susceptible maize varieties. We tested several heirloom varieties (Fig. S8), including Oaxacan Green, which has a reputation for stress tolerance. We also tested Boone County Dent (Boone), a commercially available varietal, and the research line B73 for their response to salt stress (Fig. 2C). We chose to compare Boone and B73 to the Oaxacan Green variety as it exhibited similar control primary root length at 5 days post-germination to the Boone variety (Fig. S8). All varieties showed significantly reduced root growth in response to 50 mM NaCl treatment; however, primary root growth in Oaxacan Green was significantly less sensitive to 50 mM salt treatment compared to Boone and B73 (Fig. 2C; two-way ANOVA interaction, *P*<0.0001). Oaxacan Green was also significantly less susceptible to salt stress under 100 mM NaCl conditions compared to Boone and B73 [Fig. 2C; ANOVA, *P*<0.005 (Boone versus Oaxacan Green), *P*<0.0001 (B73 versus Oaxacan Green)]. Based on these differences in salt stress responses, we proceeded to perform DESI-MSI on Oaxacan Green to map how chemical localization changes across development in a genotype with resilience to salt stress.

We applied DIMPLE to newly generated DESI-MSI data for Oaxacan Green roots at approximately 50 µm resolution. To compare the composition of mass signatures in B73 and Oaxacan Green maize varieties, we pooled the DIMPLE-processed *m/z* values (within a ±0.001 *m/z* window) for three replicates of each maize variety. There were 301 unique mass signatures identified across three B73 roots (Tables S1, S2 and S8), and 1082 unique mass signatures identified in three Oaxacan Green roots (Fig. 2D, Tables S8 and S9). The increase in mass signatures in Oaxacan Green compared to B73 is likely due to instrumentation variability over time, which is a known challenge in MSI. HPLC-MS data suggest that B73 and Oaxacan Green produce similar numbers of mass signatures, with 1110 and 1139 unique mass signatures (within a ±0.001 *m/z* window) identified, respectively, for B73 and Oaxacan Green. An initial comparison revealed that 727 of the HPLC-MS mass signatures were shared between varieties, with 383 unique to B73 and 412 unique to Oaxacan Green (Table S10). A comparison (within a ±0.001 *m*/z window) of the unique DESI-MSI mass signatures reported for B73 and Oaxacan Green identified 91 mass signatures conserved between the varieties, 210 mass signatures unique to B73 and 991 unique to Oaxacan Green (Fig. 2D, Table S8). The conserved signatures may represent a core set of metabolites in developing root tissue. The unique mass signatures identified in each variety, particularly in Oaxacan Green, are exciting candidates as they may confer different developmental responses to the environment.

To continue characterizing differences between the varieties, we compared the linescan graphs along the developmental axis of each root variety, which revealed striking differences in the developmental localization patterns of metabolites (Fig. 2A,B,E,F). B73 roots generally had four distinct clusters that align closely to developmental regions (Fig. 2B, Fig. S1), as previously described. Oaxacan Green roots, however, typically grouped into five distinct clusters, the first four being generally consistent with the B73 clusters (Fig. 2E,F, Fig. S9). In Oaxacan Green, the main clusters encompassed: Cluster 1, enrichment in the meristem and TZ; Cluster 2, distribution throughout the root axis (in one sample, the intensity was primarily meristem localized in this cluster); Cluster 3, strong signal intensity in the TZ and EZ; Cluster 4, enrichment in the mature EZ; and, finally, Cluster 5, overall low signal intensity in the TZ and EZ, with only a few pixels at normalized max intensity (Fig. S9, Tables S11-S13). In Oaxacan Green, there was more variation within each cluster, and further segmentation of these clusters could identify even more biologically meaningful localization patterns.

We focused on the 91 conserved *m/z* values identified in B73 and Oaxacan Green for our preliminary analysis of MSI data. The TCA metabolites malate, fumarate and succinate maintained general localization trends between B73 and Oaxacan Green (Figs 1D,E and 2A,B,E,F, Figs S2 and S10). Malate and fumarate both localized to the mature EZ (Fig. 2E, Figs S2 and S10) and succinate was more strongly enriched in the meristem (Fig. 2A,E, Figs S2 and S10). However, we found that several metabolites demonstrated different localization patterns in Oaxacan Green compared to B73. A noticeable difference was observed for meristem-enriched metabolites. Of the 48 meristem-localized metabolites identified in B73 (Fig. 1G), 20 were present in Oaxacan Green (Tables S3 and S8). However, around half of these shared mass signatures did not share meristem localization in Oaxacan Green (Fig. S11). For example, *m/z* 149.0118 showed specific enrichment in the root tip of B73 (Fig. 2A,B, Fig. S11); however, it was localized more broadly in Oaxacan Green roots throughout the TZ and EZ (Cluster 5) (Fig. 2E,F, Fig. S11). A similar pattern was observed for *m/z* 280.2367, which showed strong localization to the cortex in the TZ and EZ in Oaxacan Green roots [as viewed with total ion current (TIC) normalization in MSiReader] (Fig. 2E, Fig. S11). However, *m/z* 280.2364 was very specifically root tip enriched in B73 (Fig. 2A, Fig. S11). These differences do not appear to be related to root anatomy or growth, as B73 and Oaxacan Green roots have similar meristem anatomies (Fig. 2A,E). The differences in metabolic enrichment patterns between this stress-resilient and stress-susceptible variety are an exciting source of information for understanding how metabolite localization can lead to functional differences. In the next section, we will discuss a metabolite with different enrichment patterns and physiological effects in maize.

DIMPLE can rapidly identify top candidates with potential developmental significance; however, it is not designed to test the effects of different normalization approaches. By comparing DIMPLE to MSiReader visualizations, we have found that most patterns remain conserved after TIC normalization. However, it is worth noting that several *m/z* patterns change after normalization (Fig. S12), emphasizing the importance of including a normalization step before inferring biological hypotheses. The biological outcomes of these variations in metabolite localization patterns will continue to be compelling points of investigation in the future.

### D-erythrose, a compound identified through DIMPLE, improves the maize salt-stress response

**Fig. 3.**
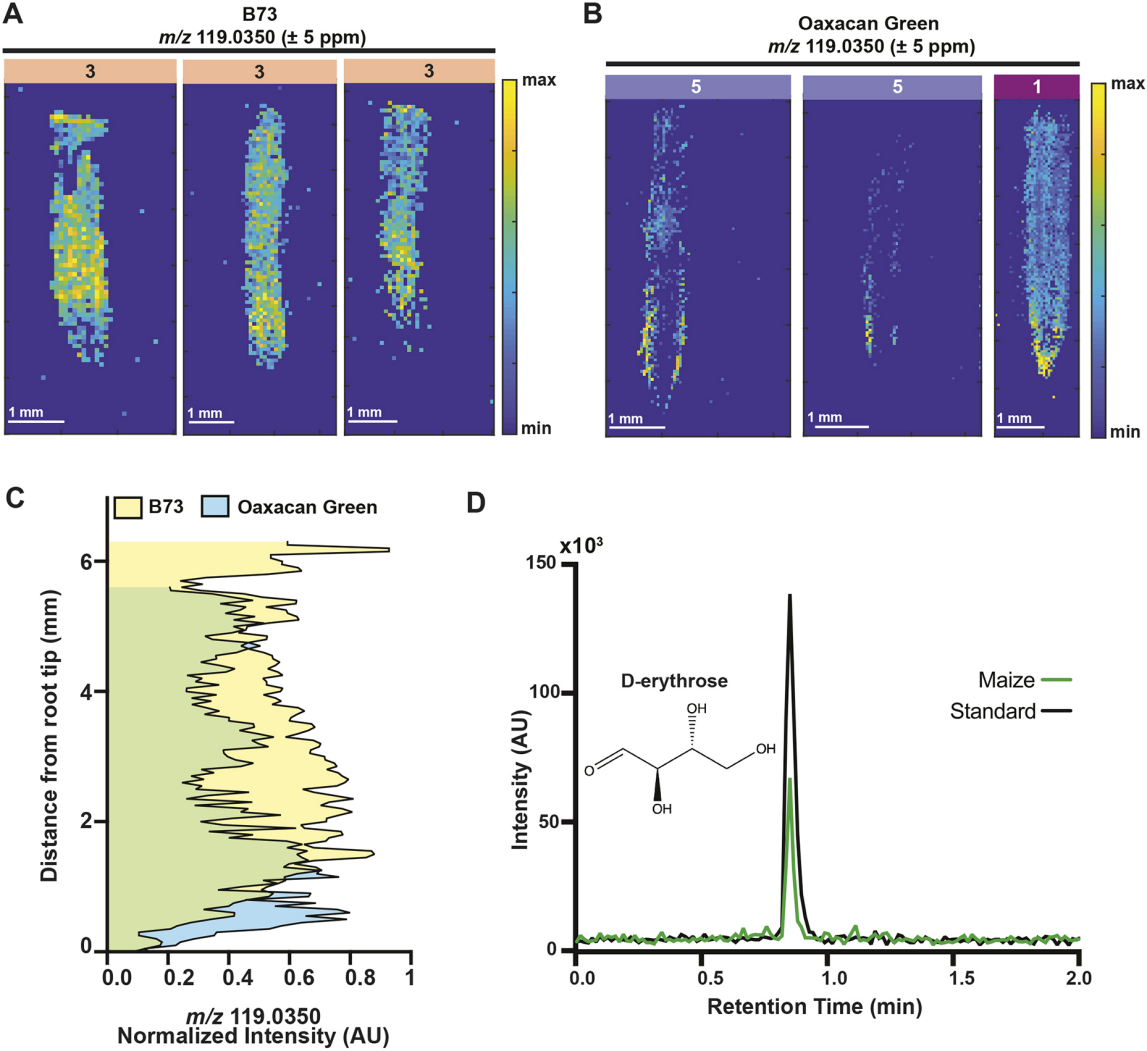
**A DIMPLE metabolite candidate with different localization patterns in B73 and Oaxacan Green is identified as erythrose.** (A) Localization pattern of *m/z* 119.0350±5 ppm grouped to Cluster 3 in three B73 replicate roots. Images are TIC normalized in MSiReader. Scale bars: 1 mm. Intensity level is represented by the colored key. (B) Localization of *m/z* 119.0350±5 ppm grouped to Cluster 5 in two biological replicates and Cluster 1 in the other replicate in three Oaxacan Green roots. Images are TIC normalized in MSiReader. Scale bars: 1 mm. Intensity level is represented by the colored key. (C) The normalized mean intensity of *m/z* 119.0350 along each 2D image of three replicate roots per variety plotted along the root axis, with blue indicating Oaxacan Green, yellow indicating B73, and green showing the overlap. (D) HPLC-MS/MS retention time for a D-erythrose standard (52 mM) (chemical structure shown) and the *m/z* 119.0350 compound in Boone maize root extract.

To determine whether metabolites with different localization patterns in B73 and Oaxacan Green influence root developmental responses to stress, we focused on a compound with an *m/z* of 119.0350, which was identified in the maize root with DIMPLE. Using DIMPLE, we found *m/z* 119.0350 localized to Cluster 3 (TZ and EZ) in B73 (Fig. 3A, Fig. S12, Tables S4-S6), and Clusters 1 (meristem) and 5 (low intensity enrichment in the TZ and EZ) in Oaxacan Green (Fig. 3B, Fig. S12, Tables S11-S13). In Oaxacan Green, the overall signal localization was present in the TZ and into the EZ but had stronger meristem enrichment and was more localized to the epidermis (Fig. S12). We visualized the *m/z* 119.0350 in MSiReader with TIC normalization: the normalized enrichment of *m/z* 119.0350 was stronger in the meristem region in Oaxacan Green and was enriched throughout the TZ and meristem in B73 (Fig. 3A-C, Fig. S12). The normalized signal was still primarily localized to the epidermis in Oaxacan Green roots and was not noticeably enriched to a specific cell layer in B73 roots. Since this is primarily a meristem- and TZ-enriched metabolite in diverse maize varieties, we hypothesized it might promote root growth.

Using multiple database searches, including Metlin, METASPACE and LOTUS, we predicted this mass signature to be D-erythrose, a tetrose monosaccharide (Fig. 3D). To validate the identity of this compound, we performed HPLC-MS/MS on Boone maize roots (Fig. 3D, Fig. S13A). DESI-MSI data showed that erythrose localizes to the Boone meristem as well (Fig. S13B). The *m/z* 119.0350 in the maize root extracts had the same MS/MS fragmentation and retention time as a D-erythrose standard, confirming that this *m/z* value likely corresponds to D-erythrose (Fig. 3D, Fig. S13A,B).

D-erythrose has been studied mostly in the context of erythrose metabolism in bacteria, yeast and animals, but has remained largely uncharacterized in plants (Andriotis and Smith, 2019; den Hartog et al., 2010; Gallardo-Pérez et al., 2023; Lee et al., 2002; Liu et al., 2015; Park et al., 2011; Williams et al., 1980; Zhang et al., 2023a). Past metabolic studies have primarily focused on erythrose-4-phosphate, an intermediate of the pentose phosphate pathway, shikimic acid pathway and the Calvin cycle (Backhausen et al., 1997; Herrmann and Weaver, 1999; Kruger and von Schaewen, 2003; Maeda and Dudareva, 2012; Sharkey, 2021). Some connections for a role of erythrose and stress response have been identified: a study in yeast found that an erythrose reductase gene is upregulated under osmotic and salt stress conditions (Park et al., 2011). Another study of salt-stressed tomato plants inoculated with and without a plant growth-promoting rhizobacterium reported that erythrose accumulated in the inoculated plants prior to stress (Mellidou et al., 2021). After salt stress, erythrose showed higher accumulation only in the non-inoculated plants (Mellidou et al., 2021).

**Fig. 4.**
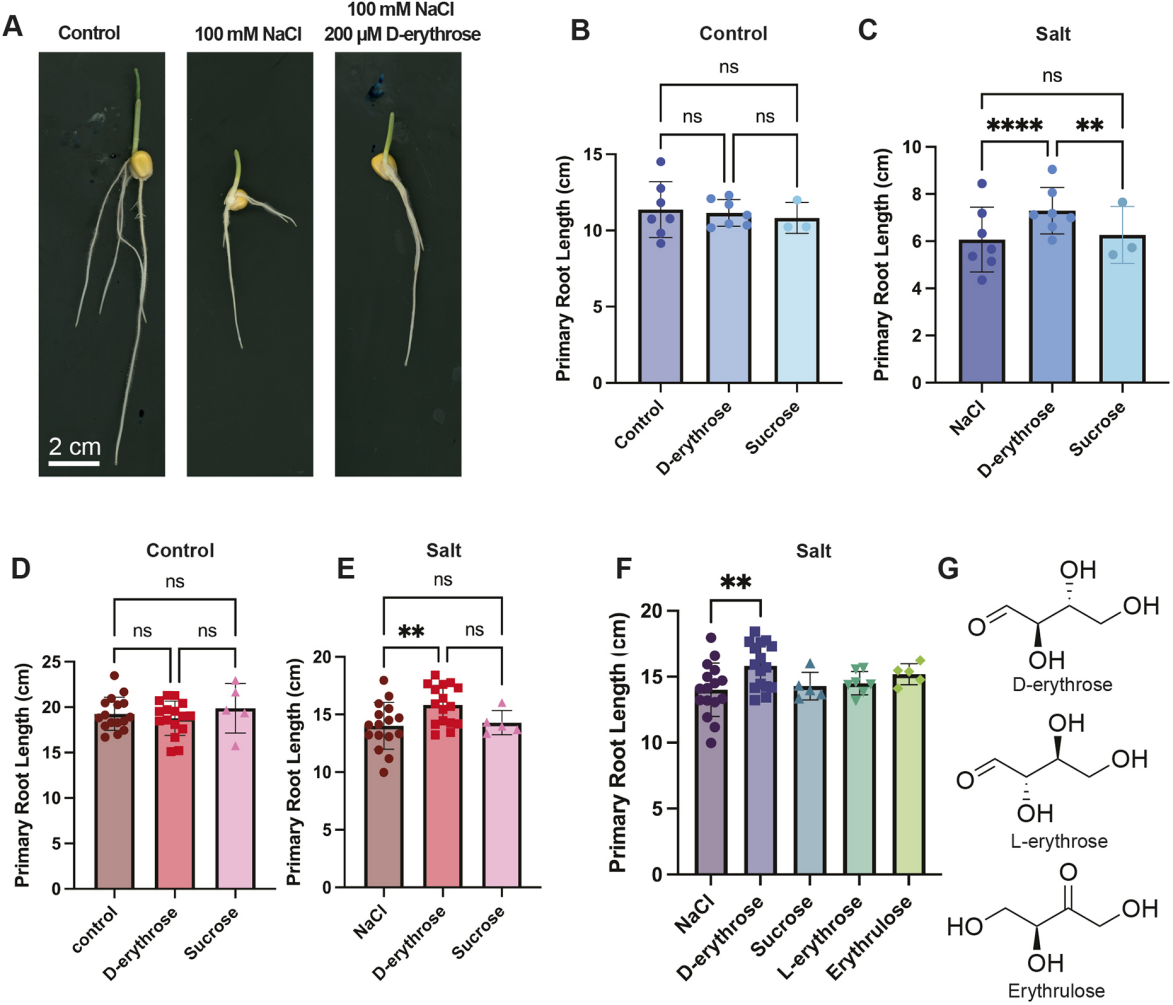
**D-erythrose, identified with DIMPLE, improves primary root growth under salt conditions.** (A) Representative images of B73 day 5 seedlings in control, 100 mM NaCl or 100 mM NaCl, and 200 µM D-erythrose treatment. Scale bar: 2 cm. (B) B73 primary root length in control conditions treated with 200 µM D-erythrose (*n*=7) or sucrose (*n*=3); control, *n*=7. Ordinary two-way ANOVA with main effects only, Tukey's multiple comparisons test, with a single pooled variance. Data reported as mean±s.d. (C) B73 primary root length in 100 mM NaCl conditions treated with 200 µM of D-erythrose (*n*=7) or sucrose (*n*=5); salt control, *n*=7. Data reported as mean±s.d. ****P<0.0001, **P=0.0065 (ordinary two-way ANOVA with main effects only, Tukey's multiple comparisons test, with a single pooled variance). (D) Boone primary root length in control conditions treated with 200 µM of D-erythrose (*n*=16) or sucrose (*n*=5); control, *n*=16. Ordinary two-way ANOVA with main effects only, Tukey's multiple comparisons with single pooled variance. Data reported as mean±s.d. (E) Boone primary root length in 50 mM NaCl conditions treated with 200 µM D-erythrose (*n*=16) or sucrose (*n*=5); control, *n*=16. Data reported as mean±s.d. **P=0.0012 (ordinary two-way ANOVA with main effects only, Tukey's multiple comparisons with single pooled variance). (F) Boone primary root lengths in 50 mM NaCl conditions treated with 200 µM D-erythrose (*n*=16), sucrose (*n*=5), L-erythrose (*n*=7) or erythrulose (*n*=5); control, *n*=16. Data reported as mean±s.d. **P=0.0023 (ordinary two-way ANOVA with main effects only, Tukey's multiple comparisons test with a single pooled variance). No other comparisons were significant. (G) Molecular structures for D-erythrose, L-erythrose and erythrulose. ns, not significant.

We performed exogenous treatments of D-erythrose on B73 and Boone combined with salt stress (Fig. 4A-E). We found no significant improvement in primary root length in control conditions with D-erythrose treatment (Fig. 4B,D). However, we found that D-erythrose treatment significantly improved Boone and B73 primary root length when combined with 50 mM NaCl and 100 mM NaCl conditions, respectively (Fig. 4A,C,E). This suggests that D-erythrose improves the salt-stress response in roots, particularly in genotypes that have increased sensitivity to stress.

To confirm that the stress response phenotype was unique to D-erythrose treatment, we treated the plants with the same concentration of sucrose, as an energy-source control. If D-erythrose is enhancing growth solely by providing additional carbohydrates to the plant, we would expect to see a similar phenotype in the sucrose-treated plants. Our results show that D-erythrose treatment significantly enhances root growth compared to sucrose-treated plants under salt conditions in B73 at 100 mM NaCl (Fig. 4C), suggesting that D-erythrose has an effect outside of supplemental energy on growth during stress. Results in Boone showed a similar trend to those in B73, with only D-erythrose capable of significantly increasing root growth during salt stress (Fig. 4D,E). To investigate whether D-erythrose may be acting as an osmolyte, or another non-specific tetrose function, we tested D-erythrose isomers, L-erythrose and erythrulose (Fig. 4F,G, Fig. S14) in salt-stressed Boone. The D-erythrose treatment was the only condition to significantly increase growth compared with salt alone (Fig. 4F). L-erythrose and erythrulose had insignificant effects compared to the control. These results may indicate a specific role for D-erythrose during salt stress.

Further investigation of the biological mechanism that controls the D-erythrose response during salt stress will reveal whether there is a functional significance to the enrichment of this compound to the meristem and TZ regions and its mechanism of stress response. The DIMPLE method provided an abundance of compounds that have not been identified or characterized for their biological significance in plant root development or stress responses. Future work to gain insight into the function of these plant molecules holds great potential to discover novel regulators of development and stress response.

### Conclusion

Overall, we have developed a computational method, DIMPLE, which leverages the linear developmental axis of plant roots to perform an untargeted screen for metabolites that have distinct developmental localization patterns. DIMPLE revealed mass signatures with specific localization to the root tip that we had not previously identified. The clustering method resulted in distinct groups corresponding generally to metabolites enriched in a variety of patterns across the developmental gradient of the B73 maize root. We then used DIMPLE to compare metabolite signatures of a salt-resilient maize variety, Oaxacan Green. We were able to identify distinct differences in the clustering between Oaxacan Green and B73, resulting in the characterization of D-erythrose, a tetrose with highly specific effects on maize roots. We found that, in salt-sensitive varieties, treatment with D-erythrose, but not other isomers, significantly improves root growth during stress.

This application of DIMPLE demonstrates that it is a useful tool for identifying uncharacterized compounds and informing biological hypotheses by providing crucial context of the developmental patterns of these compounds. DIMPLE uses hierarchical clustering to identify metabolites that share spatial patterns. The robust nature of this method results in dozens of metabolites that can continue to be characterized and investigated. For instance, DIMPLE roughly doubled the number of meristem-enriched metabolites that we were able to identify in B73. Here, we followed up on one of the many metabolites of interest that DIMPLE identified. We utilized DIMPLE to analyze maize root development, but DIMPLE can be applied to MSI data for many tissue types and research questions. It will be particularly useful for investigating metabolites that have differential enrichment patterns along a single linear axis. For example, other systems with linear developmental gradients include the proximal-distal axis of maize leaves, the crypt-villus axis in the intestine, and the antero-posterior axis in *Drosophila* embryos. Overall, DIMPLE provides an approach for reversibly converting two-dimensional MSI data to one-dimensional data for biological processes that occur along a single axis. This enables fast and statistically relevant analysis for testing specific hypotheses.

This work provides a new tool for MSI analysis, which is valuable for direct measurements of cell metabolites in their native environment. As demonstrated here, this information can inform new hypotheses of metabolite functions in development. However, a major remaining challenge is that the majority of metabolites in both plants and animals remain unannotated. Therefore, to accelerate exploration of metabolite function, it will be necessary to improve the annotation of mass signatures across biological systems. The data presented here have sufficient annotations to begin the process of investigating how the precise enrichment patterns of metabolites in the maize root inform developmental functions. Beyond the challenge of annotating mass signatures, understanding how these compounds affect development and stress responses will require further investigations. D-erythrose improved root growth under salt-stress conditions, but we have not determined the mechanism for this response. It is possible D-erythrose is metabolized to another compound that confers stress resilience, is interacting with a stress-responsive protein, or is functioning as an osmolyte. Delineating the contribution of the factors that generate the D-erythrose response will require further investigation. Additionally, it will be important to understand what processes create the D-erythrose pattern along the root developmental axis. These remaining questions for D-erythrose function and the mechanism of other annotated metabolites are an exciting avenue for future research. Future applications of DIMPLE could reveal metabolite patterns across different tissues, genotypes and environmental conditions, with broad applications in chemical biology.

## MATERIALS AND METHODS

### Maize sample preparation for DESI-MSI

The B73 samples for DESI-MSI were prepared as described by Zhang et al. (2023b). Boone and Oaxacan Green were first plated on autoclaved paper towels in a sterile 13×13 cm plate with 20 ml of DI water then transferred after 2 days and grown to 5 days in CYG Large Germination Pouches treated with 80 ml DI water. They were grown in a growth chamber set to 27°C with a 16 h:8 h day night cycle. The roots were cut at ∼5 mm from the tip, embedded in OCT and frozen on dry ice. The roots were kept at −80°C until they were removed and prepared for cryosectioning. Cryosections of 20 µm thickness were attached to microscope slides and imaged with brightfield microscopy. Each maize variety had three biological replicates prepared for MSI. The sections were packaged on dry ice and shipped to the Zare lab at Stanford University, CA, USA, for DESI-MSI. DESI-MSI experiments were performed using a commercial DESI sprayer (Viktor Tech) coupled to an Orbitrap Velos Pro mass spectrometer (Thermo Fisher Scientific). The DESI spray capillary had an inner diameter of 20 µm and an outer diameter of 150 µm. The sprayer was positioned at a 60° angle relative to the sample surface, with the capillary-to-surface distance set to 4 mm and the capillary-to-inlet distance at 2 mm. Compressed nitrogen (99.999%) was used as the nebulizing gas at 120 psi. A high voltage of −6 kV was applied to the sprayer, and the solvent (DMF:ACN, 1:1) flow rate was maintained at 0.6 µl/min. Full-scan MS data were acquired in negative ion mode over an *m/z* range of 50 to 600, with a resolution of 30,000. The scan rate was fixed by disabling automatic gain control. The maximum injection time was set at 150 µs, and the ion transfer capillary temperature was 300°C. Imaging was performed by raster-scanning the sample stage at a speed of 150 µm/s along the *x*-axis with a step size of 50 µm along the *y*-axis.

### Maize treatment experiments

For the salt sensitivity assay, B73, Boone (True Leaf Market), Oaxacan Green (David's Garden Seeds, Victory Seeds), Hopi Blue (Everwilde Farms) and Red Strawberry (Victory Seeds, Seeds Needs) were germinated on autoclaved paper towels in sterile 13×13 cm plates in either control conditions of 20 ml DI water, or 50 mM NaCl or 100 mM NaCl treatments for 2 days. The germinated seeds were transferred on day 2 into CYG Large Germination Pouches with 80 ml of the corresponding treatment. They were grown in a growth chamber set to 27°C with a 16 h:8 h day night cycle. The seedlings were grown to day 5, then scanned and primary root lengths were measured in Fiji. The sample sizes for B73, Boone and Oaxacan Green were 60 for control and 50 mM NaCl treatments, and 48 for 100 mM NaCl treatments, and 18 across all treatments for Hopi Blue and Red Strawberry. Two-way ANOVA test was performed using GraphPad PRISM.

For salt and erythrose treatments, B73, Boone (True Leaf Market) and Oaxacan Green (David's Garden Seeds, Victory Seeds) seeds were germinated on autoclaved paper towels in sterile 13×13 cm plates with corresponding 20 ml of salt (50 mM or 100 mM NaCl) and D-erythrose (Sigma-Aldrich; 200 µM) conditions, for 2 days. They were transferred on day 2 into CYG Large Germination Pouches with 80 ml of the same treatment conditions. They were grown in a growth chamber set to 27°C with a 16 h:8 h day night cycle. The plants were grown until day 5, when they were scanned, and primary root lengths were measured using Fiji. The B73 experiments without salt (Fig. 4B) had seven technical replicates for the control and D-erythrose treatments, with a total of 108 and 135 biological replicates, respectively. The B73 sucrose treatment (without salt) had three technical replicates with a total of 44 biological replicates. The B73 salt stress experiments (Fig. 4C) had seven technical replicates for the control and D-erythrose treatments, with a total of 92 and 117 total biological replicates, respectively. The B73 sucrose-salt stress experiments had three technical replicates with 46 total biological replicates. The data were analyzed in GraphPad Prism v10. All comparisons were subjected to a Shapiro–Wilk normality test prior to two-way ANOVA analysis, with all conditions passing normality except for the control-sucrose treatment, which failed (*P*=0.0086). We then performed an ordinary two-way ANOVA with main effects only and Tukey's multiple comparisons test with a single pooled variance.

The Boone experiments without salt stress (Fig. 4D) had 16 technical replicates for the control and D-erythrose treatments, with a total of 153 and 193 total biological replicates, respectively; the sucrose condition had five technical replicates with 41 total biological replicates. The Boone salt stress experiments (Fig. 4E) had 16 technical replicates for the control and D-erythrose treatments, with a total of 153 and 193 biological replicates, respectively; the sucrose condition had five technical replicates with a total of 41 biological replicates. The data were analyzed in GraphPad Prism v10. All comparisons were subjected to a Shapiro–Wilk normality test, with all conditions passing normality. We then performed an ordinary two-way ANOVA with main effects only and Tukey's multiple comparisons test with single pooled variance.

For the D-erythrose isomer treatments, Boone seeds were germinated on autoclaved paper towels in sterile 13×13 cm plates with 20 ml of salt (50 mM NaCl) and 200 µM D-erythrose (Sigma-Aldrich), sucrose, L-erythrulose (Santa Cruz Biotechnology) or L-erythrose (Santa Cruz Biotechnology) conditions. The seedlings were transferred on day 2 into CYG Large Germination Pouches with 80 ml of the same treatment conditions. They were grown in a growth chamber set to 27°C with a 16 h:8 h day night cycle. They were grown until day 5, when the primary roots were scanned and measured using Fiji. The L-erythrose and erythrulose treatments in the salt-free conditions (Fig. S14) had seven and five technical replicates and 70 and 56 biological replicates, respectively. The control, D-erythrose and sucrose replicates are as reported above. The L-erythrose and erythrulose treatments in the salt conditions (Fig. 4F) had seven and five technical replicates with 72 and 45 total biological replicates, respectively. The replicates for control, D-erythrose and sucrose are as reported above. The data were analyzed in GraphPad Prism v10. All comparisons were subjected to a Shapiro–Wilk normality test with all conditions passing normality. We then performed an ordinary two-way ANOVA with main effects only and Tukey's multiple comparisons test with a single pooled variance.

### ESI-HPLC-MS/MS identification of D-erythrose

Detection and identification of a D-erythrose standard (52 mM) in maize roots was performed using electrospray ionization high-performance liquid chromatography tandem mass spectrometry (ESI-HPLC-MS/MS). Boone County Dent maize was germinated as described above. At day 5, the primary root meristems (0.5 cm in length) were harvested. Three excised root tissues were transferred to microcentrifuge tubes with 500 µl of HPLC graded water and ground with a plastic pestle. Samples were shaken for 1 h using an analog vortex mixer (Ohaus) then centrifuged at 2800 rpm (700 ***g***) for 5 min. The extract was then filtered through a 0.2-μm syringe filter into a clean 1.5 ml tube. Finally, 100 µl of the filtrate was added to HPLC sample vial for HPLC-MS analysis. All tissue extracts were prepared on the same day. Chromatographic separation was performed on a Thermo Scientific Vanquish UHPLC system coupled to an Orbitrap Elite mass spectrometer (Thermo Scientific) equipped with a heated electrospray ionization (HESI) source. A Phenomenex Luna^®^ polar C18 column (100×2.1 mm, 1.6 µm particle size) was used for separation. The mobile phases were mobile phases A (H2O/FA, 1000/0.1, v/v) and B (ACN/FA, 1000/0.1, v/v) with the gradient program: 0-2 min, 0% B; 2-5 min, 0% B to 50% B; 5-7 min, 50% B to 99% B; 7-10 min, 99% B; 10-10.1 min, 99% B to 0% B; 10.1-13 min, 0% B. MS parameters included sheath gas flow rate of 50 arbitrary units (AU), auxiliary gas flow rate of 20 AU, spray voltage of −3.5 kV, capillary temperature of 300°C, S-lens RF level of 62.4, and resolution of 30,000. The mass spectrometer was operated in negative ionization mode with data collected in centroid format. A targeted MS/MS method was used with two scan events. Both scan events used higher-energy collisional dissociation fragmentation with a normalized collision energy of 35%, a maximum injection time of 100 ms, and an isolation window of 1.0 *m/z*. The first scan event was centered on a precursor at *m/z* 119.00, with fragment ions collected across an *m/z* range of 50-125. The second scan event was centered on a precursor at *m/z* 101.00, with fragment ions collected across an *m/z* range of 50-115. In both cases, the precursor charge state was set to 1, with no compensation voltage applied. Data acquisition and method control were performed using Thermo Xcalibur software.

### ESI-HPLC-MS/MS identification of metabolite features in B73 and Oaxacan Green

At 5 days post germination, primary root tips (0.5 cm) were harvested from B73 and Oaxacan Green maize grown as described above in DI water. Sample preparation was conducted as described in the above section. Chromatographic separation was performed on a Thermo Scientific Vanquish UHPLC system coupled to an Orbitrap Elite mass spectrometer (Thermo Scientific) equipped with a HESI source. A Phenomenex Kinetex^®^ C18 reverse-phase column (150×2.1 mm, 1.7 µm particle size) was used for separation. The mobile phases were mobile phase A (H2O/FA, 1000/0.1, v/v) and mobile phase B (ACN/FA, 1000/0.1, v/v) with the following gradient program: 0-1 min, 0% B; 1-9 min, 0% B to 100% B; 9-11 min, 100% B; 11-11.5 min, 100% B to 0% B; 11.5-14 min, 0% B. The flow rate was maintained at 0.4 ml/min, with a total run time of 14 min. MS parameters included a spray voltage of −3.5 kV, sheath gas flow rate of 50 arbitrary units (AU), auxiliary gas flow rate of 20 AU, capillary temperature of 300°C, S-lens RF level of 69.1 and resolution of 30,000. The mass spectrometer was operated in negative ionization mode, and data were collected in centroid format. A data-dependent top six scan MS/MS method was employed with seven scan events per duty cycle. The resulting MS/MS spectra were acquired at a resolution of 30,000. Data acquisition and method control were performed using Thermo Xcalibur software.

### R code

R was used to compile a list of all the peaks present across three biological replicates. Unique peaks were defined within 0.001 tolerance of each other. The compiled lists for B73 and Oaxacan Green were compared to identify shared and unique peaks between the two groups. The code was used to identify peaks for DIMPLE-generated and ESI-HPLC-MS/MS data. Certain aspects of the code were written with assistance from ChatGPT; the AI tool provided suggestions that were evaluated and tested to achieve the final analysis. The full R code analysis can be found in the Dickinson Lab Github at https://github.com/dickinsonlab.

### Technical aspects of DIMPLE

Source code and raw data for DIMPLE are available on the Dickinson Lab GitHub (https://github.com/dickinsonlab) and at https://zenodo.org/records/17187822.

MSI data of each tissue section consists of a series of .raw files with each file containing the mass spectrum for a full row of pixels. To import MSI data into MATLAB, we converted the .raw files to .cdf using Xcalibur 3.0 Thermo File Converter. We then used the self-written MATLAB function ‘Batchcdfread’, provided by the Zare lab, to read the data with the appropriate number of .cdf files specified. This function is linked in the GitHub repository for the DIMPLE code. Each file contains one row of pixels that is imported into a nested MATLAB cell array by batchcdfread and reorganized into a 2D layout corresponding to the image. A sum-projection is calculated for each pixel and used to draw a mask for the tissue area of interest. Everything outside the masked region is considered background. Once a mask is selected, all peaks in each spectrum (pixel) are identified and assigned to *m/z* values based on the highest intensity for each peak. An array is then prepared with the summed intensity values corresponding to the center *m/z* value. A median filter is then applied to the summed spectrum to reduce noise and create a cleaner spectrum to detect meaningful features in the bulk spectrum. The filtered data are then downsampled to a 2.5 ppm tolerance window to identify significant peaks and avoid conflicts between closely spaced *m/z* values. The tolerance window can be adjusted to fit the user's instrument acquisition parameters. The background peaks, defined as peaks present outside of the mask, are similarly filtered and prepared. The sample peaks are compared to the background peaks and filtered depending on whether the sample intensity is greater than the minimum fold enrichment of the background peaks, for our analysis this was set to 1.5. This final set of peaks thus corresponds to matching center *m/z* positions across the entire image. The data are reorganized into a hyperspectral image in which each ‘channel’ corresponds to the intensity distribution across the image at each unique *m/z* value.

The DIMPLE script includes code for exporting the hyperspectral image in a TIFF container for visualization and analysis using standard image processing tools such as ImageJ. DIMPLE itself performs simple analyses of the spatial distribution of signal in each channel. First, channels that are only sparsely present in a subset of pixels are removed using morphological erosion as channels where all pixels with >0 intensity are exclusively surrounded by 0-valued pixels will be removed.

For the analysis of developmental patterns in plant roots, a linescan is generated by performing a maximum projection along the lateral axis of the root. For pattern detection, each linescan is normalized to a maximum intensity of 1. The linescan data are then hierarchically clustered using Ward's method with a Euclidean norm as the pair-wise measure of pattern similarity, to a defined number of cluster groups selected after inspecting the resulting dendrogram shape. The linescans in each cluster can be visualized with the linescan graph or using the linescan GUI. The GUI enables annotation of features in the linescan graph and recovers the 2D image for individual linescans. These can be exported as images.

Certain aspects of the code, mainly pertaining to the visualization of the data, were written with assistance from ChatGPT. The AI tool provided MATLAB code that was then edited and reformatted to achieve the desired visualizations of the data.

## Supplementary Material

10.1242/develop.205350_sup1 Supplementary information

Table S1. Zhang et al. 2023 vs DIMPLE *m/z* lists. Comparison of the peaks identified in the Zhang et al. 2023 paper and the unique *m/z* values identified across three replicate B73 roots using DIMPLE. B73 replicates were pooled and the unique values were identified as peaks within ± 0.001 *m/z*. Red highlighted peaks were not identified using DIMPLE.

Table S2. B73 replicate peaklists. The DIMPLE processed final peaks identified in each B73 replicate root.

Table S3. Meristem Comparisons. Comparison between the root tip enriched peaks identified in the Zhang et al. 2023 paper and the peaks that group to DIMPLE Cluster 1 across three B73 replicates. The blue highlighted peaks are ones that were not included in cluster 1 but grouped to different DIMPLE clusters. The red highlighted peaks were not identified using DIMPLE.

Table S4. Annotated Clusters in B73 Root 1. The list and highlighted cluster assignment for each peak detected in B73 root 1 using DIMPLE.

Table S5. Annotated Clusters in B73 Root 2. The list and highlighted cluster assignment for each peak detected in B73 root 2 using DIMPLE.

Table S6. Annotated Clusters in B73 Root 3. The list and highlighted cluster assignment for each peak detected in B73 root 3 using DIMPLE.

Table S7. Analysis of *m/z* with specific localization patterns. Annotations of *m/z* values with observed specificity to either the cortex, or vasculature or showing a distinct bimodal pattern. Values are highlighted to represent how many replicates this pattern was observed in, 1 (yellow), 2 (blue) or 3 (green).

Table S8. Comparison of *m/z* values from DIMPLE in Oaxacan Green and B73. Unique and conserved *m/z* values for three replicates of Oaxacan Green, and three replicates of B73 roots were analyzed and compared within a ± 0.001 *m/z* threshold.

Table S9. Oaxacan Green replicates peaklist. The DIMPLE processed final peaks identified in each B73 replicate root.

Table S10. Comparison of *m/z* values from HPLC-MS in Oaxacan Green and B73. Unique and conserved *m/z* values for a Oaxacan Green and a B73 root tip were analyzed and compared within a ± 0.001 *m/z* threshold.

Table S11. Annotated Clusters in Oaxacan Green Root 1. The list and highlighted cluster assignment for each peak detected in Oaxacan Green root 1 using DIMPLE.

Table S12. Annotated Clusters in Oaxacan Green Root 2. The list and highlighted cluster assignment for each peak detected in Oaxacan Green root 2 using DIMPLE.

Table S13. Annotated Clusters in Oaxacan Green Root 3. The list and highlighted cluster assignment for each peak detected in Oaxacan Green root 3 using DIMPLE.
