# Supplementary material for: Computational method to analyze linear developmental gradients reveals specific metabolite enrichment patterns in stress-tolerant maize
Source: Development. 2026 May 7;153(8):dev205350. doi: 10.1242/dev.205350 (PMC13200719; doi:10.1242/dev.205350)
Supplement: Supplementary information [file develop-153-205350-s1.pdf]

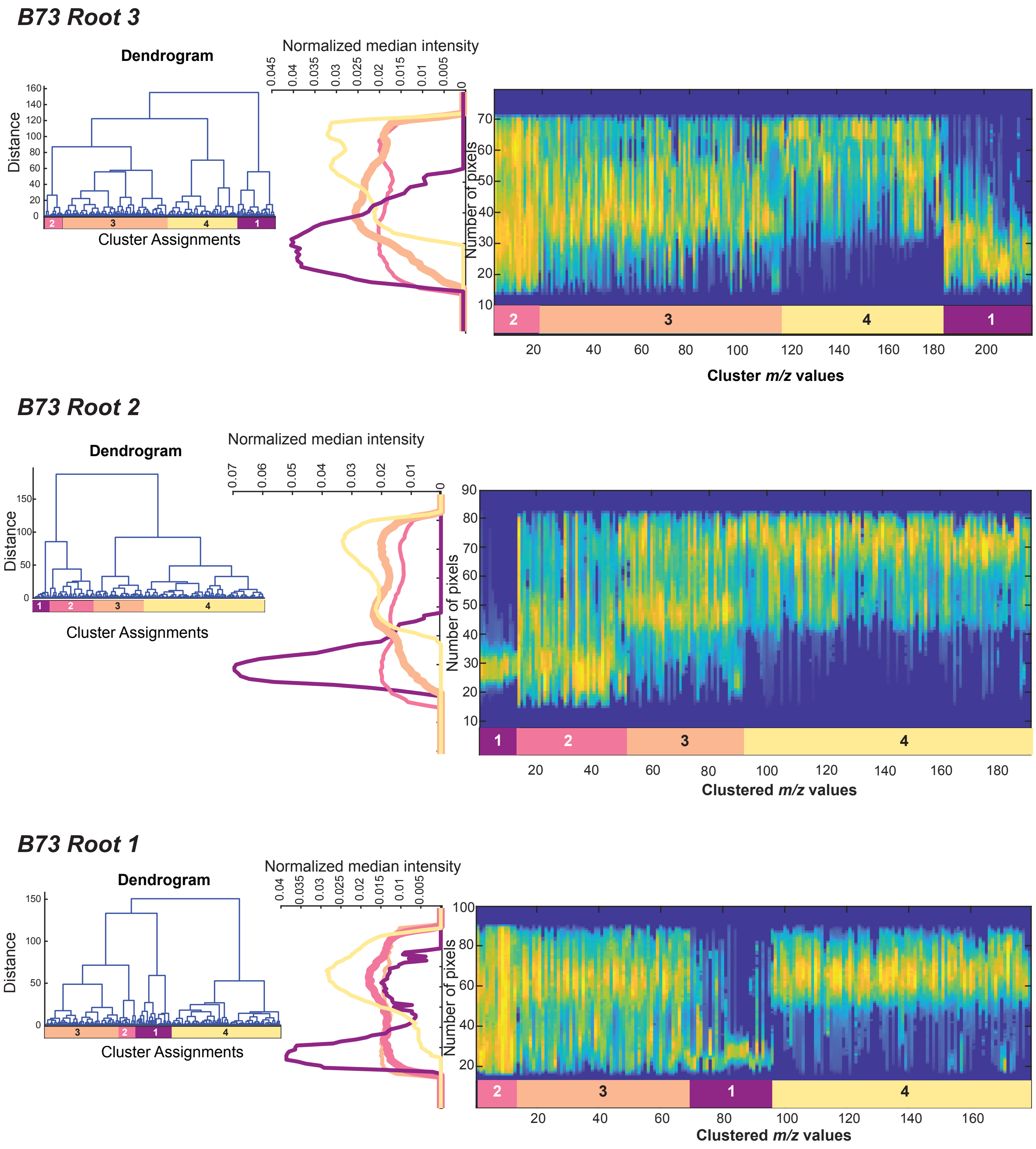

Fig. S1. Dendrograms and linescan graphs for three B73 root replicates. Dendrograms use Ward's method clustering to identify four main clusters. Linescan graphs showing the intensity profiles of mass signatures in each cluster. The y-axis is the position along the root axis where the root tip is at the origin. The x-axis corresponds to the clustered *m/z* linescans arranged smallest to largest within each cluster. The intensity of each linescan is normalized to a maximum value of 1. The intensity plots next to the linescan show the normalized median linescan intensity for each cluster along the root axis.

Development • Supplementary information

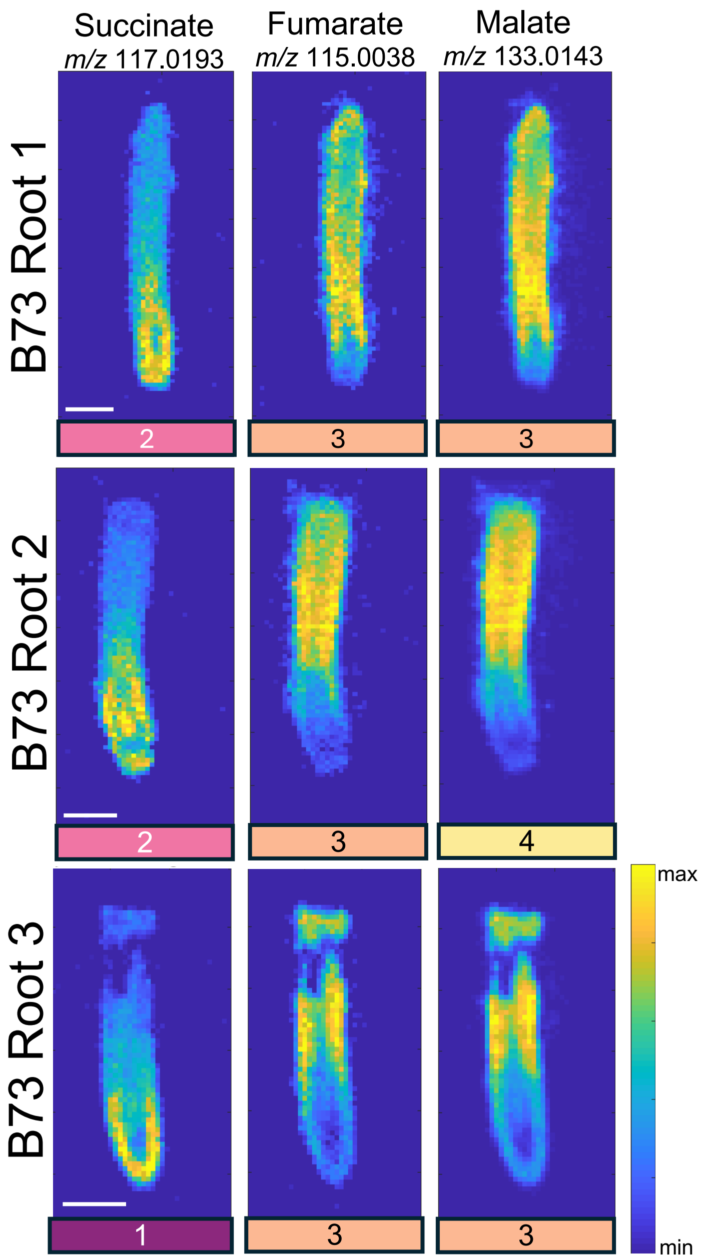

Fig. S2. TCA metabolites, succinate, fumarate and malate. MSI patterns from three B73 roots are shown. TIC-normalized with MSiReader +/- 5 ppm. Scale bar = 1 mm.

Development • Supplementary information

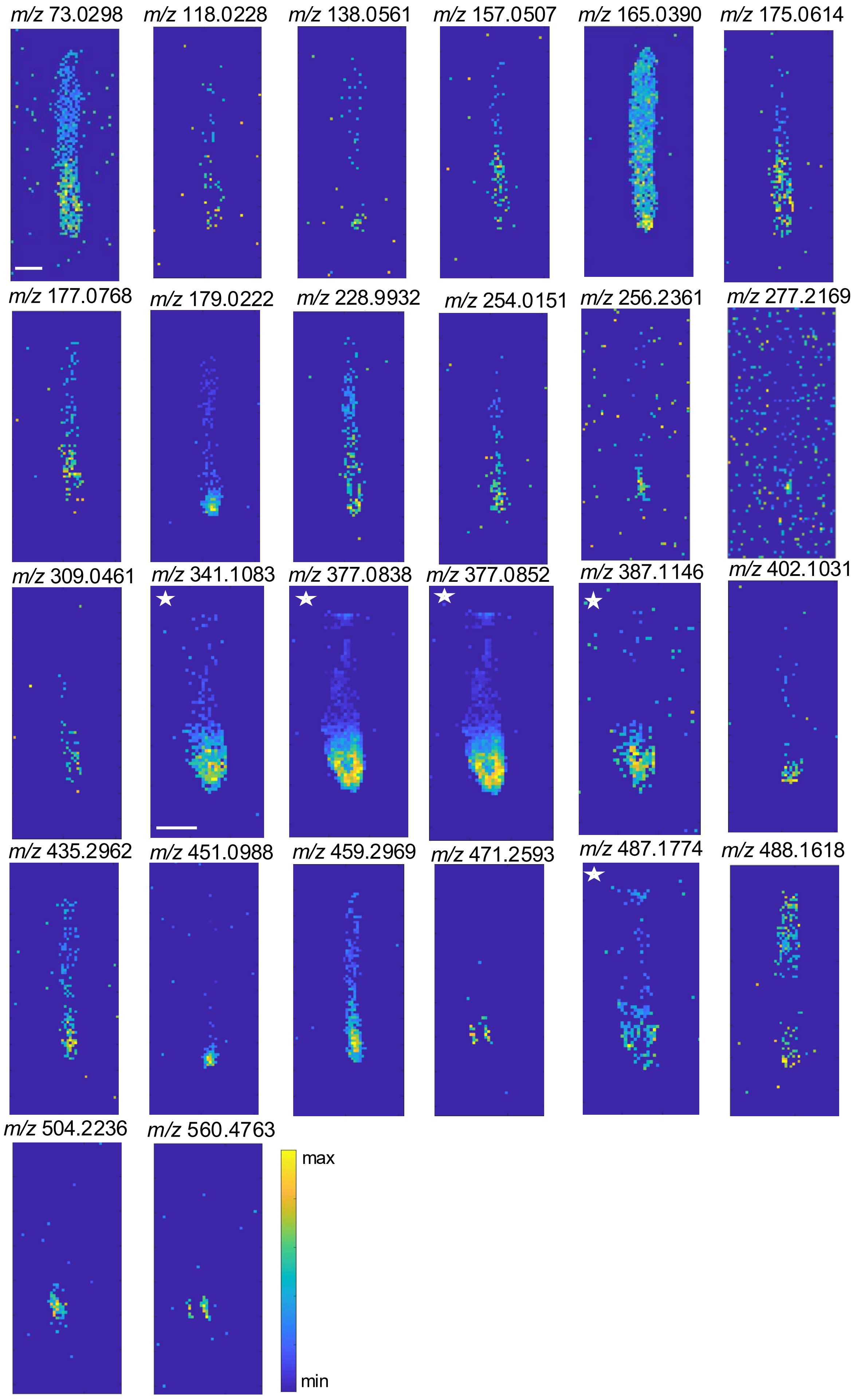

Fig. S3. New mass signatures identified with DIMPLE Cluster 1 characterized by meristem localization. MSI patterns from B73 root 1 (unstarred) and B73 root 3 (starred) are shown. TIC normalized in MSiReader, +/- 5 ppm. Scale bar = 1 mm.

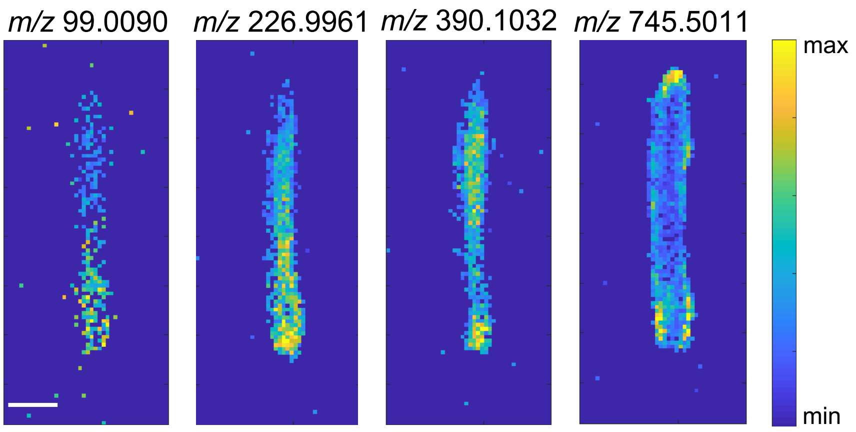

Fig. S4. Mass signatures with meristem enrichment identified in Zhang et al. 2023 that did not localize to DIMPLE Cluster 1. MSI patterns from B73 root 1 shown. TIC normalized in MSiReader +/- 5 ppm. Scale bar = 1 mm.

Development • Supplementary information

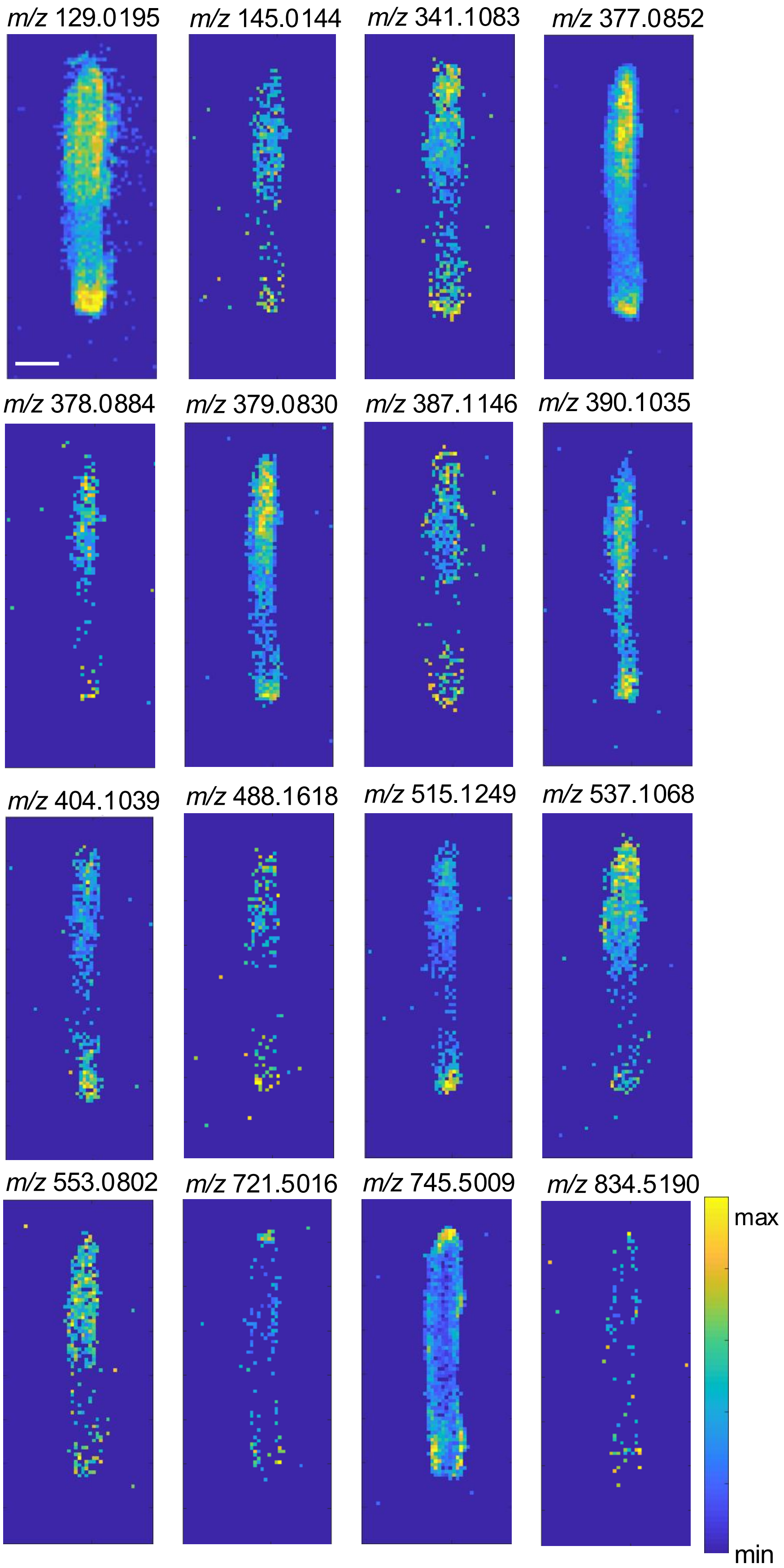

Fig. S5. Subset of bimodal distribution patterns observed in the DIMPLE GUI. MSI patterns from B73 root 1 is shown. TIC normalized with MSiReader +/- 5 ppm. Scale bar = 1 mm.

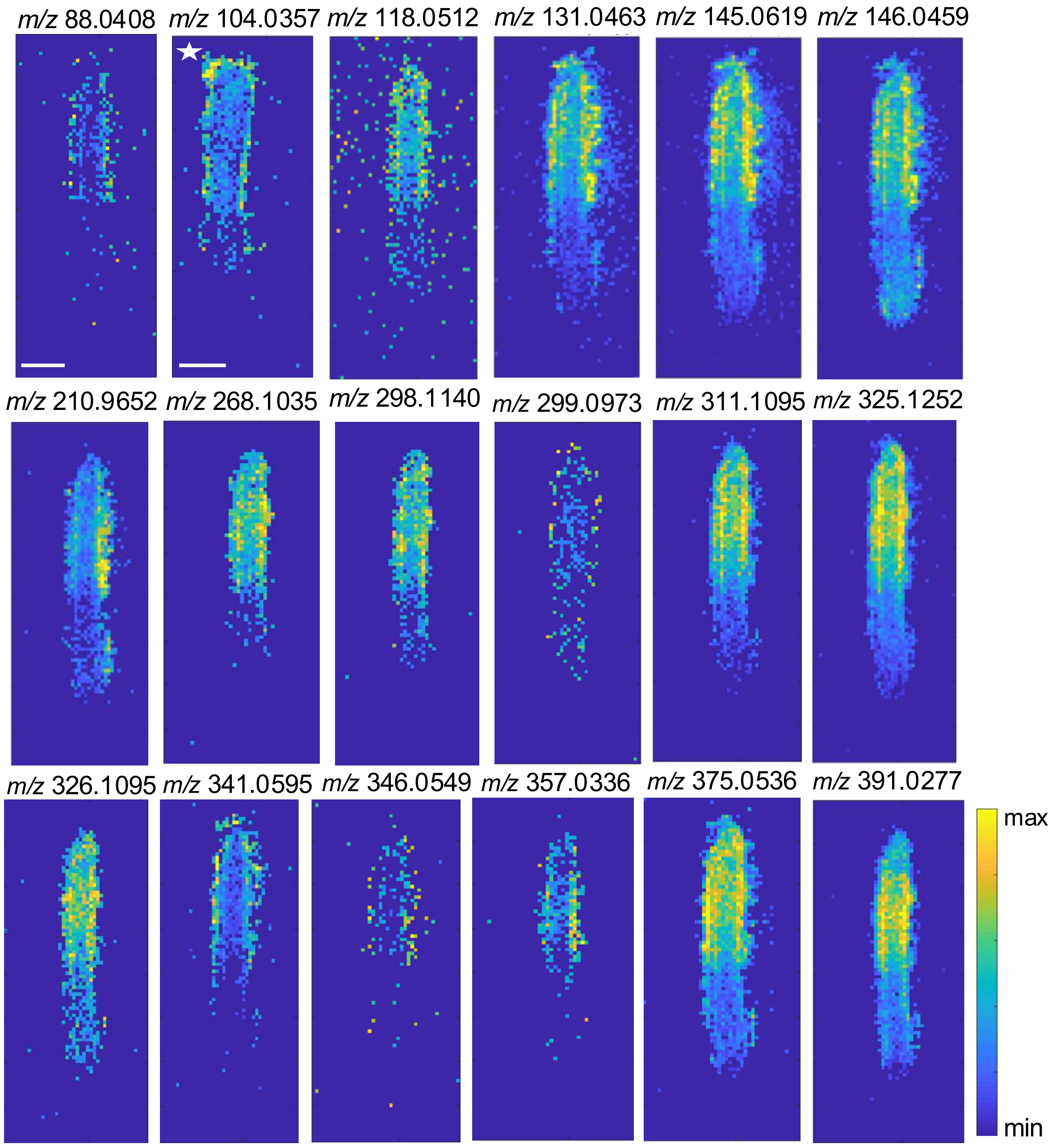

Fig. S6. Subset of cortex localized mass signatures identified with the DIMPLE GUI, MSI patterns from B73 root 1 (unstarred) and B73 root 2 (starred) are shown. TIC normalized with MSiReader +/- 5 ppm. Scale bar = 1 mm.

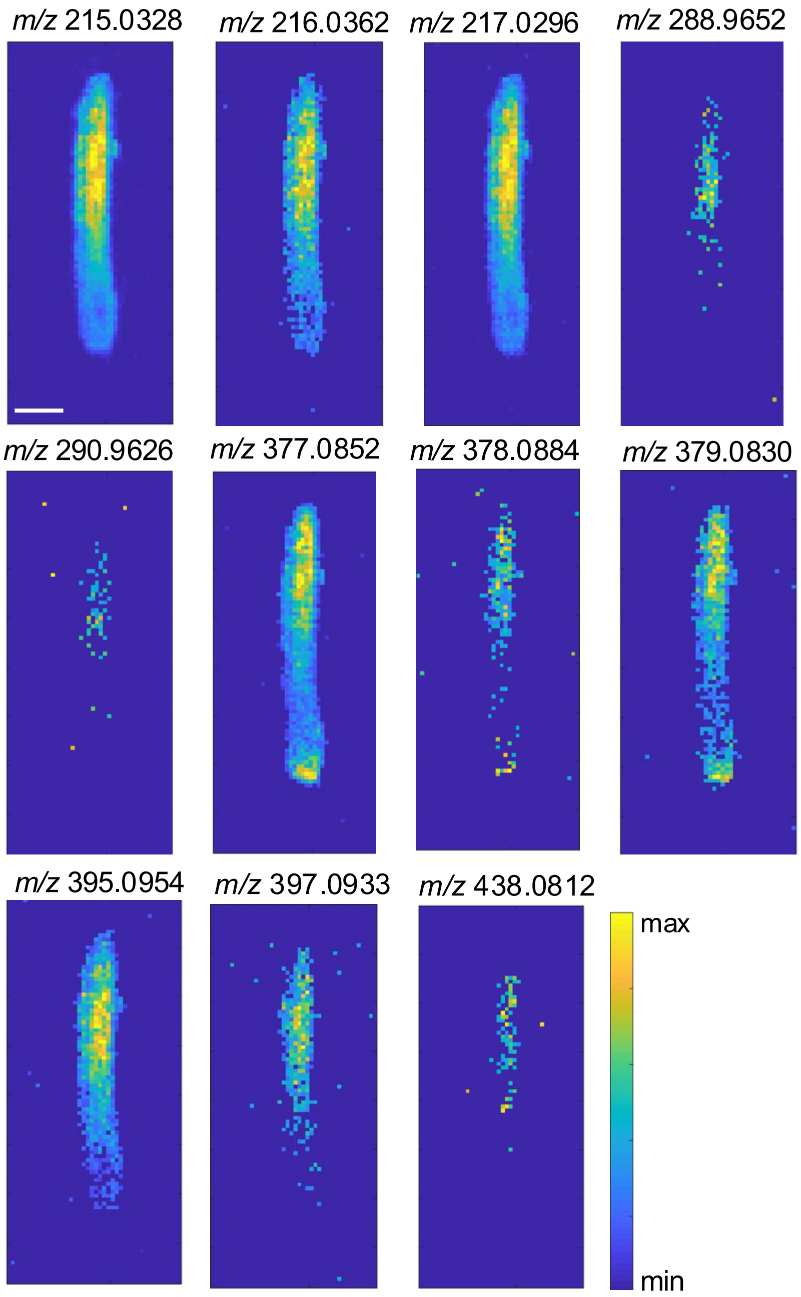

Fig. S7. Subset of vasculature localized mass signatures identified with DIMPLE GUI. MSI patterns from B73 root 1 is shown. TIC normalized with MSiReader +/- 5 ppm. Scale bar = 1 mm.

Development • Supplementary information

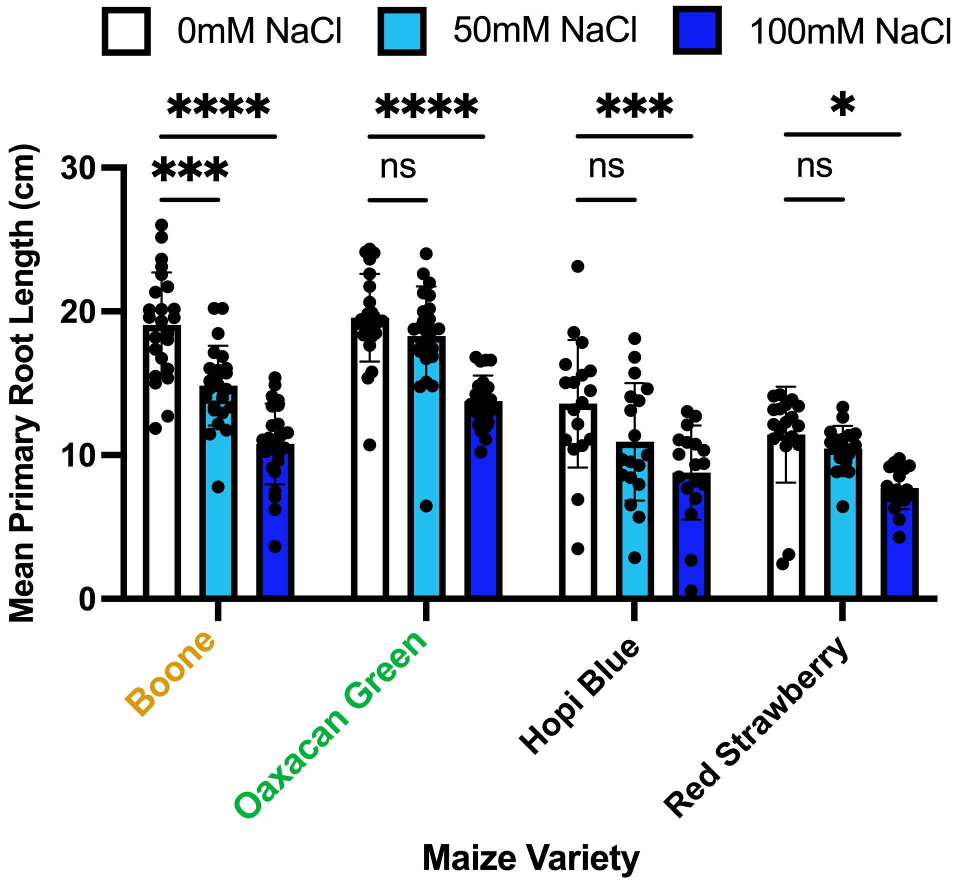

Fig. S8. Heirloom maize varieties Oaxacan Green, Hopi Blue and Red Strawberry were more salt resilient compared to Boone County Dent maize. (50 mM and 100 mM NaCl). Maize was treated with either control, 50 mM NaCl or 100 mM NaCl conditions for 5 days. The primary root length was measured for each condition. 2-way ANOVA, Tukey's multiple comparisions. Sample size for Boone n=24, Oaxacan Green n=24, Hopi Blue n=18, Red Strawberry n=18. ****P<0.0001, ***P<0.001, *P<0.05.

Development • Supplementary information

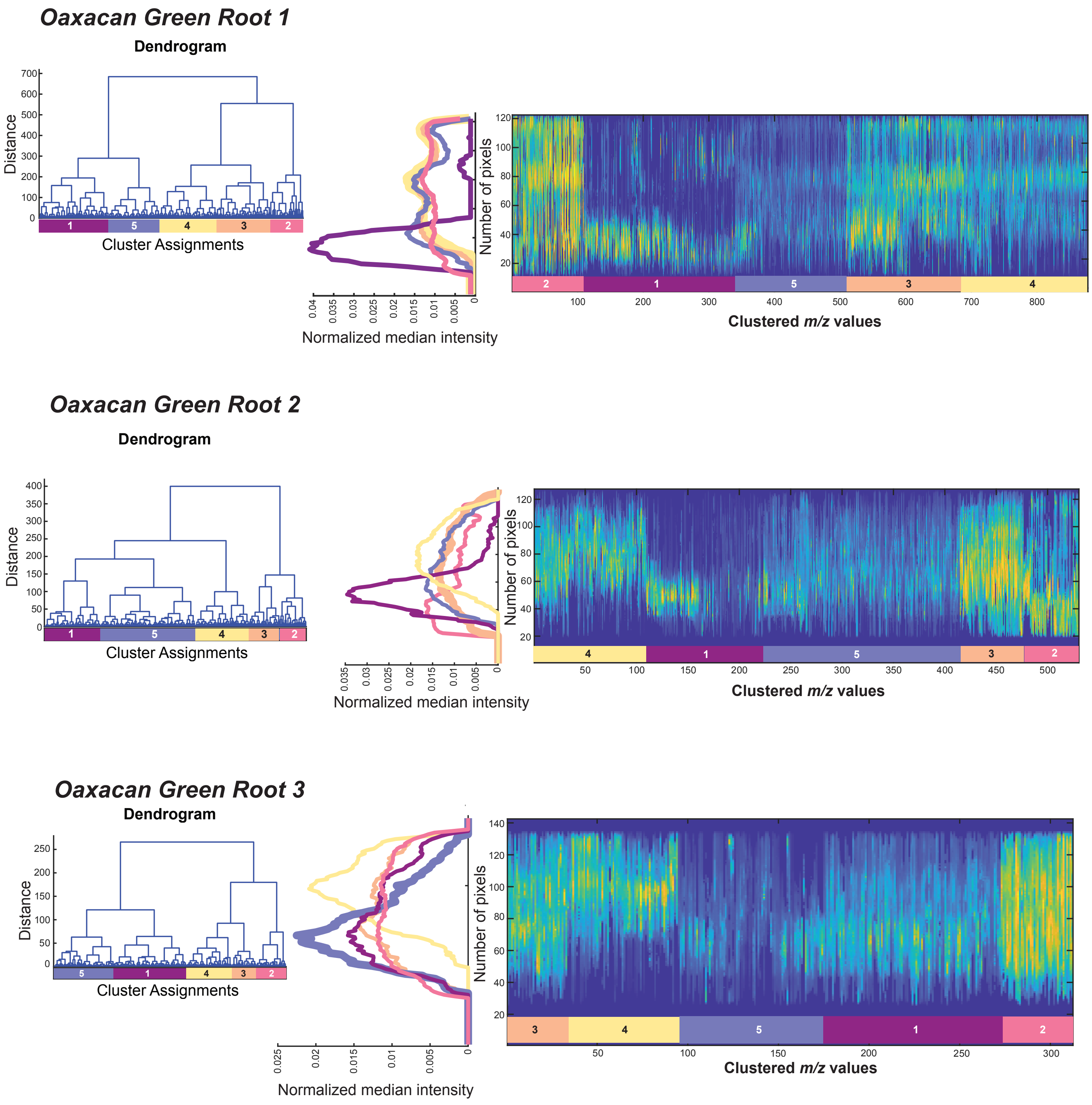

Fig. S9. Dendrograms and linescans for three replicate Oaxacan Green roots. Dendrograms use Ward's method clustering to identify five main clusters. Linescan graphs show the intensity profiles of mass signatures in each cluster. The y-axis is the position along the root axis where the root tip is at the origin. The x-axis corresponds to the clustered *m/z* linescans arranged smallest to largest within each cluster. The intensity of each linescan is normalized to a maximum value of 1. The intensity plots next to each linescan show the normalized median linescan intensity for each cluster.

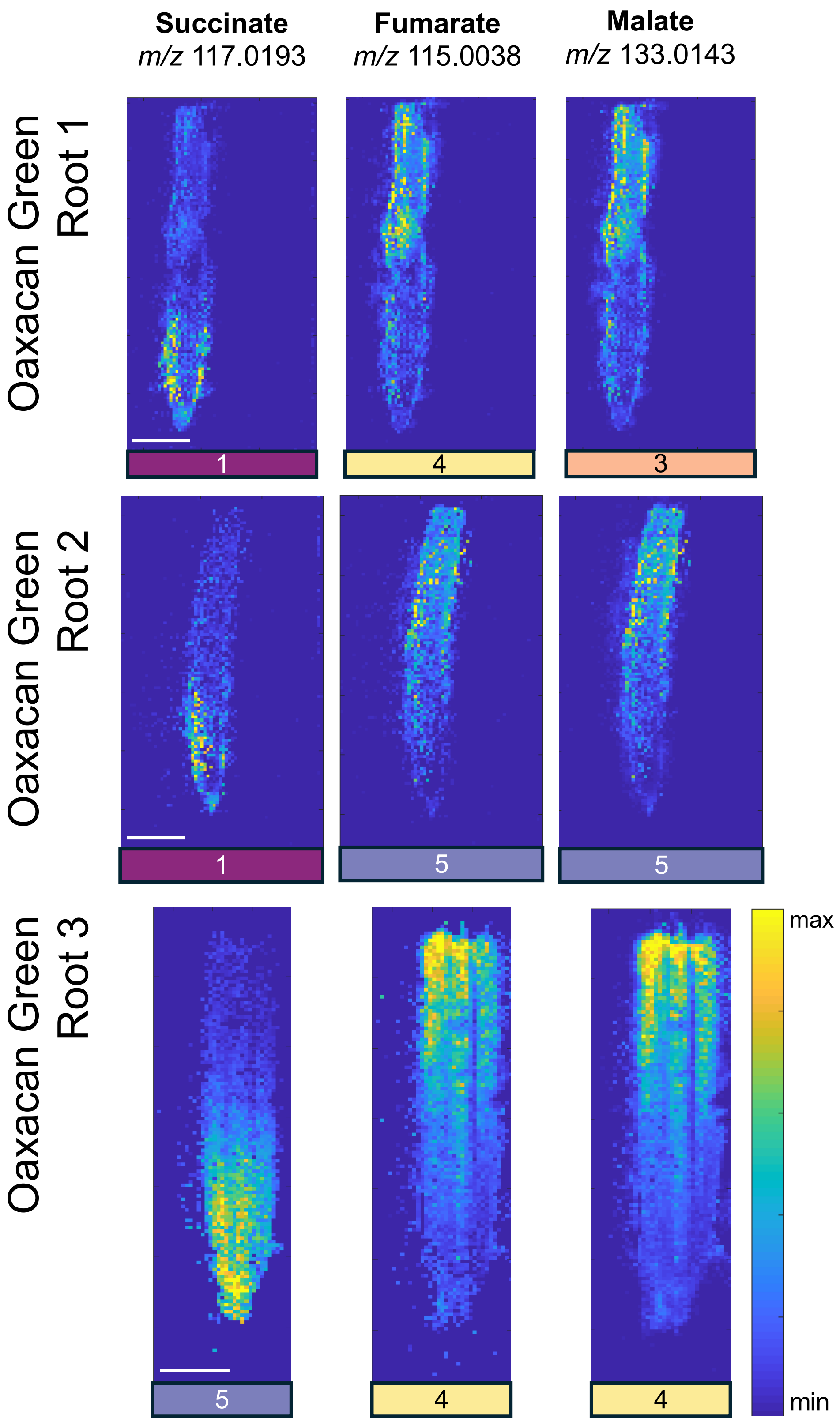

Fig. S10. TCA metabolites, succinate, fumarate and malate. MSI patterns from three Oaxacan Green roots. TIC-normalized metabolites with MSiReader, +/- 5 ppm. Scale bar = 1 mm.

Development • Supplementary information

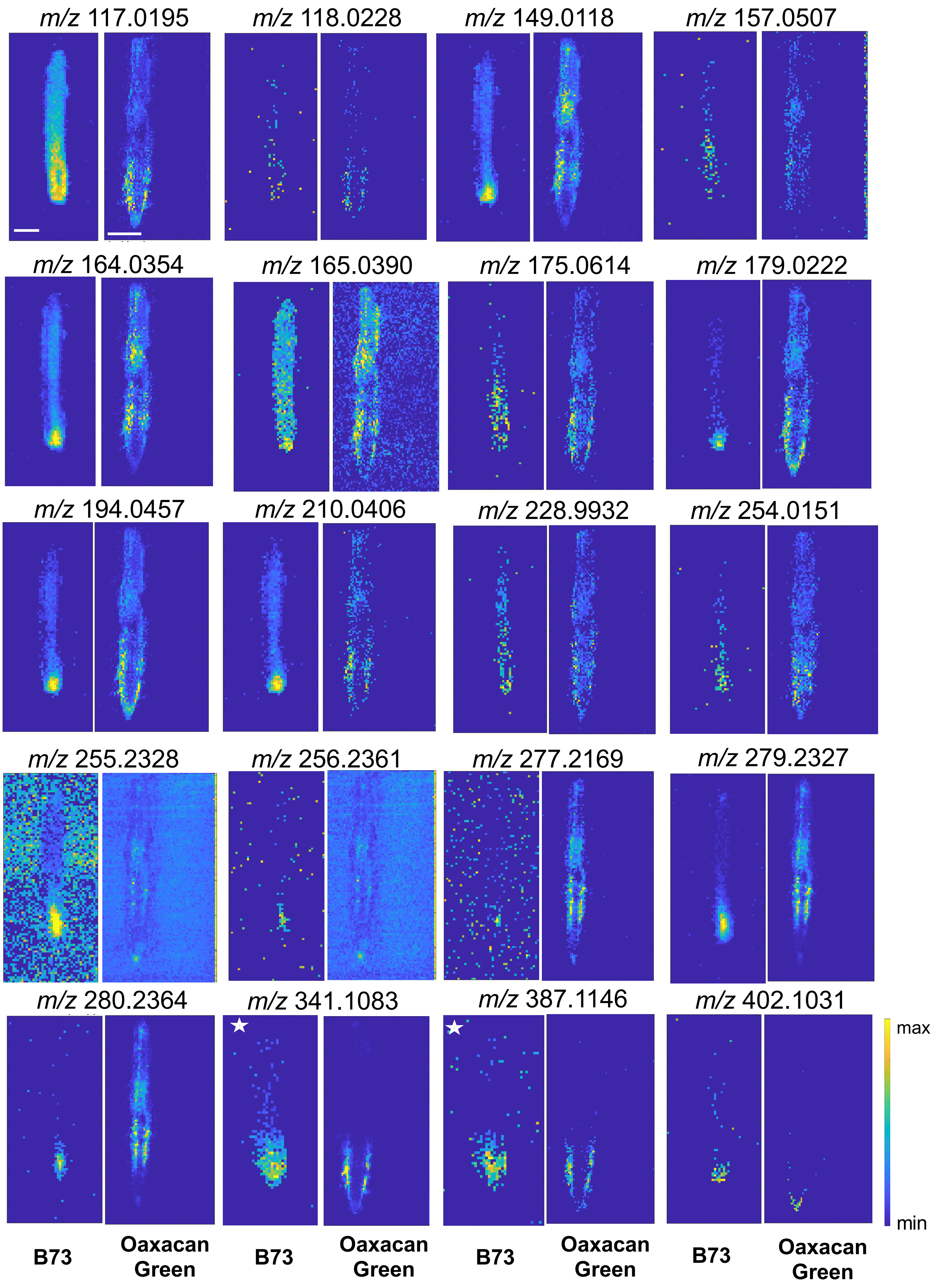

Fig. S11. Comparison of meristem enriched compounds identified in B73 and their localization in Oaxacan Green. 20 meristem enriched metabolites in B73 were present in the Oaxacan Green data, several show different localization patterns. MSI patterns from B73 root 1 (unstarred), and B73 root 3 (starred) and Oaxacan Green root 1 are shown. TIC normalized with MSiReader, +/- 5 ppm. Scale bar = 1 mm.

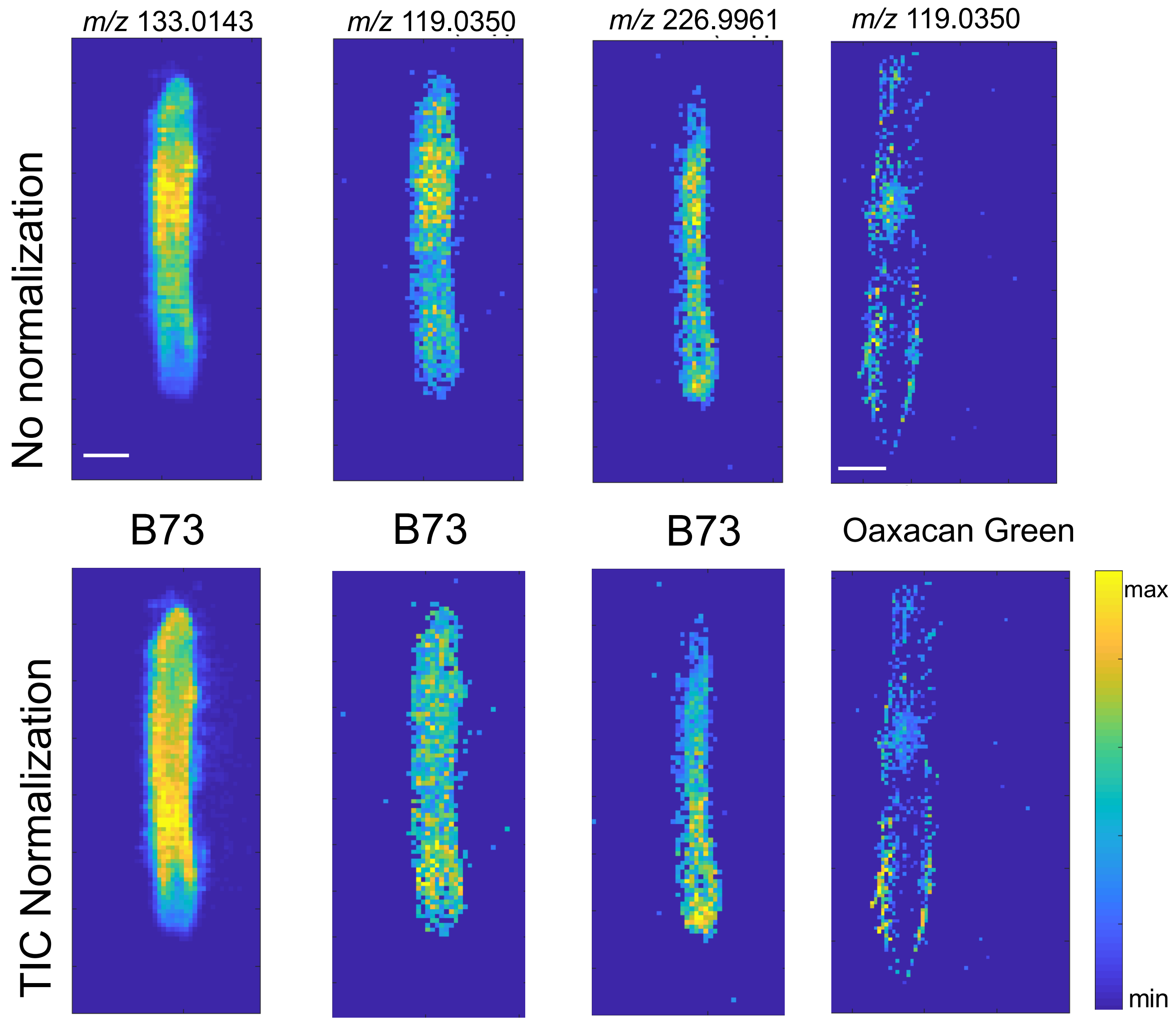

Fig. S12. Shifts in metabolite localization with TIC normalization. B73 and Oaxacan Green root metabolites with and without TIC normalization, MSI patterns from B73 root 1 and Oaxacan Green root 1 are shown. Generated in MSiReader +/- 5 ppm. Scale bar = 1 mm.

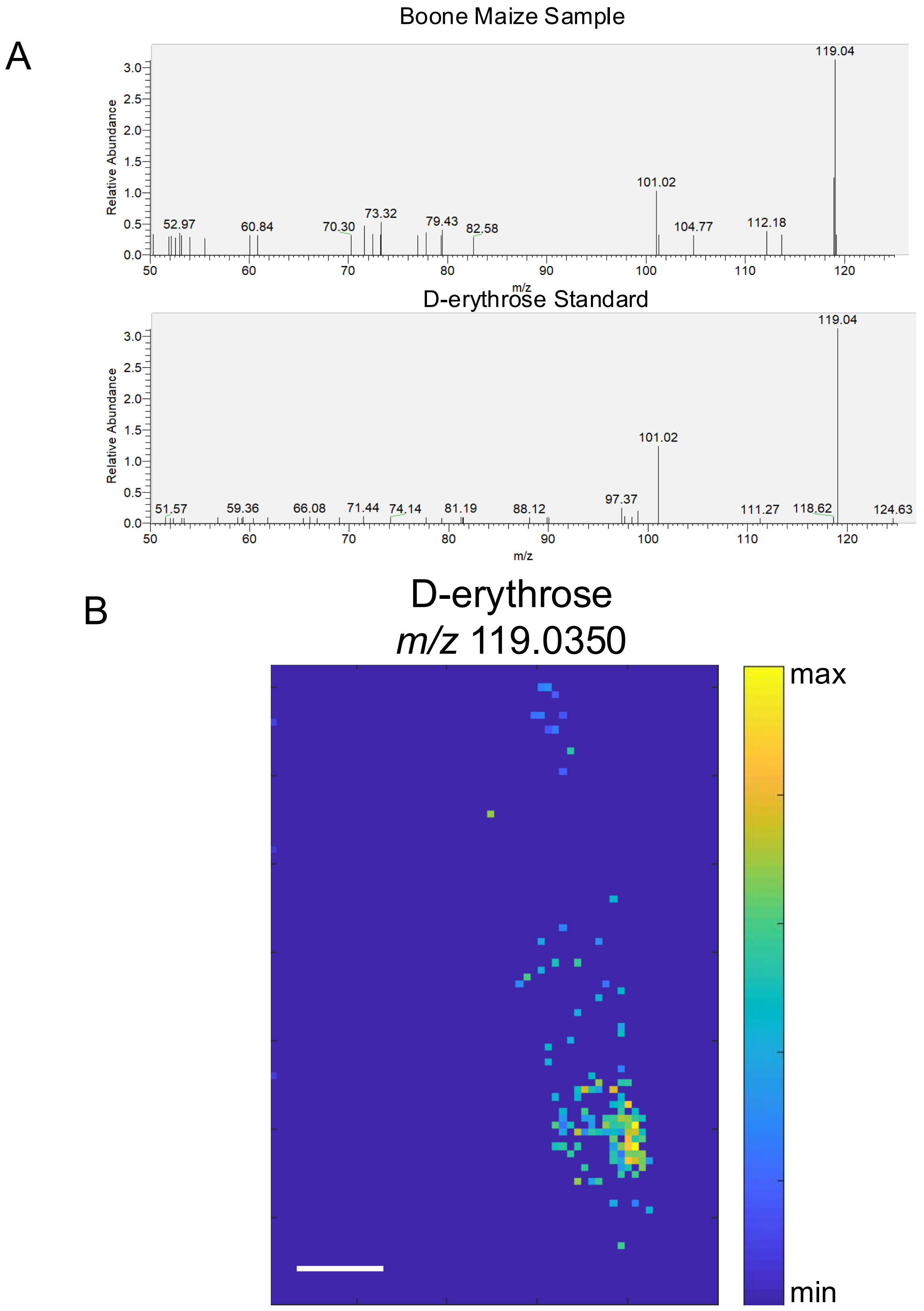

Fig. S13. A) HPLC-MS/MS fragmentation patterns for 119.0350 parent ion into *m/z* 101.02 in Boone County Dent maize roots and an erythrose standard (52 mM) visualized in Xcalibur. B) DESI-MSI of erythrose signature in a Boone root. TIC normalized with MSiReader +/- 5 ppm. Scale bar = 1 mm.

Development • Supplementary information

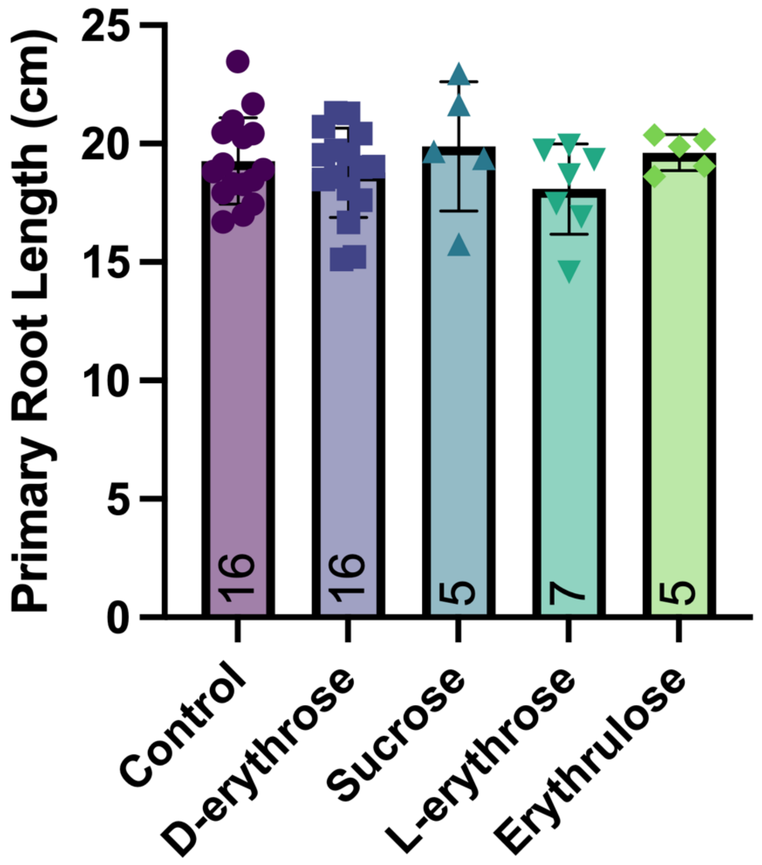

Fig. S14. Boone primary root length treated with D-erythrose isomers under control conditions. Ordinary two-way ANOVA with main effects only, Tukey's multiple comparisons test, with a single pooled variance. Sample sizes for each treatment: Control N = 16, D-erythrose N = 16, Sucrose N = 5, L-erythrose N = 7, Erythrulose N = 5.

Table S1. Zhang et al. 2023 vs DIMPLE *m/z* lists. Comparison of the peaks identified in the Zhang et al. 2023 paper and the unique *m/z* values identified across three replicate B73 roots using DIMPLE. B73 replicates were pooled and the unique values were identified as peaks within ± 0.001 *m/z*. Red highlighted peaks were not identified using DIMPLE.

Available for download at
https://journals.biologists.com/dev/article-lookup/doi/10.1242/dev.205350#supplementary-data

Table S2. B73 replicate peaklists. The DIMPLE processed final peaks identified in each B73 replicate root.

Available for download at
https://journals.biologists.com/dev/article-lookup/doi/10.1242/dev.205350#supplementary-data

Table S3. Meristem Comparisons. Comparison between the root tip enriched peaks identified in the Zhang et al. 2023 paper and the peaks that group to DIMPLE Cluster 1 across three B73 replicates. The blue highlighted peaks are ones that were not included in cluster 1 but grouped to different DIMPLE clusters. The red highlighted peaks were not identified using DIMPLE.

Available for download at
https://journals.biologists.com/dev/article-lookup/doi/10.1242/dev.205350#supplementary-data

Table S4. Annotated Clusters in B73 Root 1. The list and highlighted cluster assignment for each peak detected in B73 root 1 using DIMPLE.

Available for download at
https://journals.biologists.com/dev/article-lookup/doi/10.1242/dev.205350#supplementary-data

Table S5. Annotated Clusters in B73 Root 2. The list and highlighted cluster assignment for each peak detected in B73 root 2 using DIMPLE.

Available for download at
https://journals.biologists.com/dev/article-lookup/doi/10.1242/dev.205350#supplementary-data

Table S6. Annotated Clusters in B73 Root 3. The list and highlighted cluster assignment for each peak detected in B73 root 3 using DIMPLE.

Available for download at
https://journals.biologists.com/dev/article-lookup/doi/10.1242/dev.205350#supplementary-data

Table S7. Analysis of *m/z* with specific localization patterns. Annotations of *m/z* values with observed specificity to either the cortex, or vasculature or showing a distinct bimodal pattern. Values are highlighted to represent how many replicates this pattern was observed in, 1 (yellow), 2 (blue) or 3 (green).

Available for download at
https://journals.biologists.com/dev/article-lookup/doi/10.1242/dev.205350#supplementary-data

Table S8. Comparison of *m/z* values from DIMPLE in Oaxacan Green and B73. Unique and conserved m/z values for three replicates of Oaxacan Green, and three replicates of B73 roots were analyzed and compared within a ± 0.001 *m/z* threshold.

Available for download at
https://journals.biologists.com/dev/article-lookup/doi/10.1242/dev.205350#supplementary-data

Table S9. Oaxacan Green replicates peaklist. The DIMPLE processed final peaks identified in each B73 replicate root.

Available for download at
https://journals.biologists.com/dev/article-lookup/doi/10.1242/dev.205350#supplementary-data

Table S10. Comparison of *m/z* values from HPLC-MS in Oaxacan Green and B73. Unique and conserved *m/z* values for a Oaxacan Green and a B73 root tip were analyzed and compared within a ± 0.001 *m/z* threshold.

Available for download at
https://journals.biologists.com/dev/article-lookup/doi/10.1242/dev.205350#supplementary-data

Table S11. Annotated Clusters in Oaxacan Green Root 1. The list and highlighted cluster assignment for each peak detected in Oaxacan Green root 1 using DIMPLE.

Available for download at
https://journals.biologists.com/dev/article-lookup/doi/10.1242/dev.205350#supplementary-data

Table S12. Annotated Clusters in Oaxacan Green Root 2. The list and highlighted cluster assignment for each peak detected in Oaxacan Green root 2 using DIMPLE.

Available for download at
https://journals.biologists.com/dev/article-lookup/doi/10.1242/dev.205350#supplementary-data

Table S13. Annotated Clusters in Oaxacan Green Root 3. The list and highlighted cluster assignment for each peak detected in Oaxacan Green root 3 using DIMPLE.

Available for download at
https://journals.biologists.com/dev/article-lookup/doi/10.1242/dev.205350#supplementary-data
